# Disruption of the Key Ca^2+^ Binding Site in the Selectivity Filter of Neuronal Voltage-Gated Calcium Channels Inhibits Channel Trafficking

**DOI:** 10.1016/j.celrep.2019.08.079

**Published:** 2019-10-01

**Authors:** James O. Meyer, Shehrazade Dahimene, Karen M. Page, Laurent Ferron, Ivan Kadurin, Joseph I.J. Ellaway, Pengxiang Zhao, Tarun Patel, Simon W. Rothwell, Peipeng Lin, Wendy S. Pratt, Annette C. Dolphin

**Affiliations:** 1 Department of Neuroscience, Physiology and Pharmacology, University College London, London WC1E 6BT, UK

**Keywords:** N-type, P/Q-type, calcium channel, selectivity filter, α_2_δ subunit, β subunit, divalent cation, permeation, trafficking, calcium currents

## Abstract

Voltage-gated calcium channels are exquisitely Ca^2+^ selective, conferred primarily by four conserved pore-loop glutamate residues contributing to the selectivity filter. There has been little previous work directly measuring whether the trafficking of calcium channels requires their ability to bind Ca^2+^ in the selectivity filter or to conduct Ca^2+^. Here, we examine trafficking of neuronal Ca_V_2.1 and 2.2 channels with mutations in their selectivity filter and find reduced trafficking to the cell surface in cell lines. Furthermore, in hippocampal neurons, there is reduced trafficking to the somatic plasma membrane, into neurites, and to presynaptic terminals. However, the Ca_V_2.2 selectivity filter mutants are still influenced by auxiliary α_2_δ subunits and, albeit to a reduced extent, by β subunits, indicating the channels are not grossly misfolded. Our results indicate that Ca^2+^ binding in the pore of Ca_V_2 channels may promote their correct trafficking, in combination with auxiliary subunits. Furthermore, physiological studies utilizing selectivity filter mutant Ca_V_ channels should be interpreted with caution.

## Highlights

• Selectivity filter mutations in Ca_V_2 channels block inward Ba^2+^ currents
• Surprisingly, these mutations severely reduce trafficking of the Ca_V_2 channels
• Pore mutant N-type channels show reduced expression in presynaptic terminals
• Pore mutant channels still require β and α_2_δ and thus are not grossly misfolded

## Graphical Abstract

**Figure:**
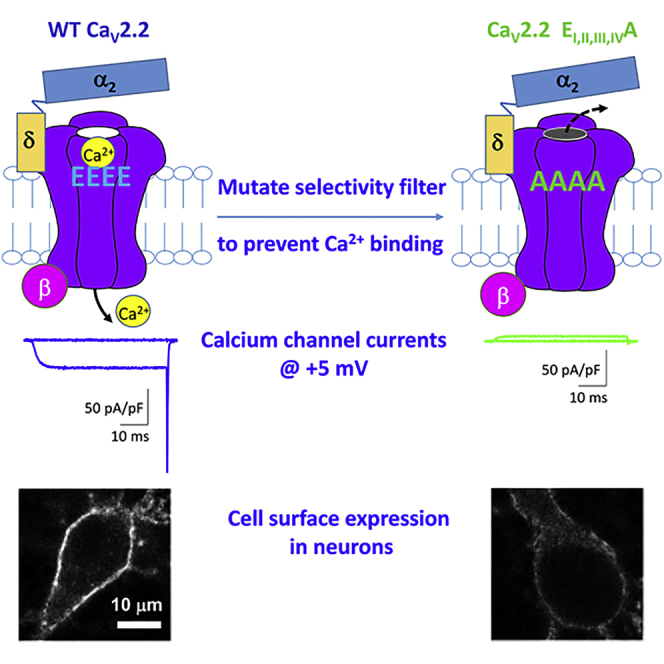

## Teaser

Meyer et al. examine whether selectivity filtering mutations in Ca_V_2 channels, preventing inward Ba^2+^ currents, would reduce trafficking. Pore-mutant channels show strongly reduced cell-surface expression in cell lines and neurons, but still require β and α_2_δ subunits and thus are not grossly misfolded.

## Introduction

Voltage-gated calcium channels (Ca_V_) are exquisitely Ca^2+^ selective (Hess and Tsien, 1984), and the molecular basis for this key attribute was probed as soon as the first L-type channels were cloned. The α_1_ subunits form the core of the channels and determine their main biophysical and pharmacological properties (Catterall, 2011, Zamponi et al., 2015). The pore was identified by homology to other voltage-gated channels as being formed from transmembrane segments 5 and 6 and the pore-lining loop between them in each of the four homologous domains (Tanabe et al., 1987). Initial seminal studies with Ca_V_1.2 identified the fundamental role of four conserved selectivity filter glutamates (E) for divalent cation binding in the channel pore and for permeation (Ellinor et al., 1995, Yang et al., 1993). Key evidence for their involvement in the Ca^2+^ binding sites within the pore came from the finding that they are essential to divalent cation-mediated block of channel permeation by monovalent cations. Indeed, these studies supported the idea of a single high (μM)-affinity binding site that is also occupied by blocking cations such as Cd^2+^ (Yang et al., 1993).

Elucidation of the nature of the channel pore selectivity filter came from the crystal structure of a bacterial channel formed by mutating the tetrameric sodium channel, NavAb, so that the pore became Ca^2+^ selective, producing CavAb (Tang et al., 2014). This revealed three sequential Ca^2+^ binding sites in the pore, the middle one having the highest affinity for Ca^2+^, dependent on four carboxyl side chains from the acidic pore-lining residues. This site is also occupied by blocking cations and is equivalent to the high-affinity site identified in mammalian Ca_V_ channels (Yang et al., 1993), whose orientation is confirmed in the cryoelectron microscopy (cryo-EM) structure of Ca_V_1.1 (Wu et al., 2016).

For mammalian Ca_V_ channels containing mutations in their selectivity filter E residues, there has been little previous work directly measuring whether their trafficking to the plasma membrane or into neuronal processes is compromised by their inability to bind or conduct Ca^2+^. In the early studies on Ca_V_1.2, the presence of an outward current was taken as an indicator that the selectivity filter mutant channels reached the plasma membrane (Ellinor et al., 1995, Yang et al., 1993). Following this work, similar selectivity filter mutant channels have since been utilized in several functional studies to examine the pre- and post-synaptic consequences of expressing divalent cation-impermeant calcium channels. In these studies, the expectation was that such channels would act as dominant negatives by occupying specific limiting sites in the plasma membrane (Cao et al., 2004, Cao and Tsien, 2010, Krey et al., 2013).

In the present study, we tested the ability of Ca_V_2 selectivity filter mutant channels to reach the plasma membrane in both cell lines and hippocampal neurons and to extend into the processes and presynaptic terminals of these neurons. We correlated the function of these channels to their trafficking, utilizing exofacially tagged Ca_V_2 channels that report cell-surface expression (Cassidy et al., 2014). Our aims were (1) to determine whether the selectivity filter mutant channels had intrinsic trafficking defects and (2) to determine whether these related to their ability to interact with the calcium channel auxiliary α_2_δ and β subunits. These auxiliary subunits are essential for correct Ca_V_1 and Ca_V_2 channel function and trafficking in both neurons and cell lines (Cassidy et al., 2014, Gurnett et al., 1996, Kadurin et al., 2016, Waithe et al., 2011); therefore, if they had an effect on the selectivity filter mutant channels, this would indicate that the channels were correctly folded. The β subunits increase Ca_V_ currents by binding to the α-interaction domain (AID) on the intracellular I-II linker (Pragnell et al., 1994) and promoting its α-helix formation (Van Petegem et al., 2004). In contrast, the mechanism is less clear whereby the α_2_δ subunits increase trafficking of the channel complex (Cantí et al., 2005, Cassidy et al., 2014) and promote voltage-dependent activation (Kadurin et al., 2016, Savalli et al., 2016). The recent structure of the skeletal muscle Ca_V_1.1 complex (Wu et al., 2016) elucidates a complex interaction among several extracellular loops in domains I–III of Ca_V_1.1 with α_2_δ-1 domains, including the von Willebrand factor (VWA) domain, as also elucidated functionally (Bourdin et al., 2017, Cantí et al., 2005, Dahimene et al., 2018).

Our main finding is that there are substantial defects in trafficking to the cell surface and into neuronal processes and presynaptic terminals of the divalent cation-impermeant Ca_V_2 selectivity filter mutant channels, even though they are optimally expressed together with the auxiliary β and α_2_δ subunits. Furthermore, cell-surface expression of these selectivity filter mutant channels was still elevated by the auxiliary subunits. However, the effect of the β subunit was consistently reduced, suggesting it may act in concert with Ca^2+^ binding in the pore to promote the correct folding of the channel in the endoplasmic reticulum (ER). Our results suggest that a combination of interaction with the auxiliary subunits and Ca^2+^ binding in the pore promotes the trafficking competency of Ca_V_2 channels.

## Results

### Ca_V_2.2 Channels with Mutations in Their Selectivity Filters Have Reduced or No Inward Calcium Channel Current

**Figure 1:**
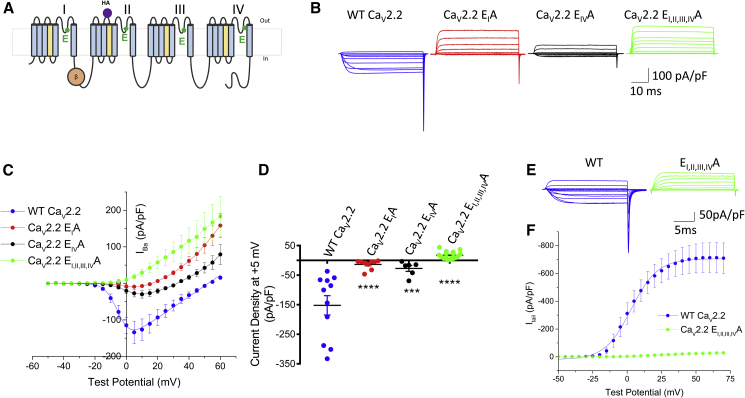
Calcium Channel Currents Produced by Selectivity Filter Mutant Ca_V_2.2 Channels in Comparison to WT Channels (A) Diagram of Ca_V_2.2 with the positions of the HA tag in domain II and the four selectivity filter glutamates (E residues) indicated. All Ca_V_2.2 constructs contained this HA tag, unless stated otherwise. (B) Example families of Ca_V_2.2 currents recorded from tsA-201 cells for WT Ca_V_2.2-HA (blue), Ca_V_2.2-HA E_I_A (red), Ca_V_2.2-HA E_IV_A (black), and Ca_V_2.2-HA E_I, II, III, IV_A (green), co-expressed with β1b and α_2_δ-1. Holding potential −80 mV, steps between −50 and +60 mV for 50 ms in 5-mV steps. (C) Mean (±SEM) *I-V* relationships for the conditions shown in (B). WT Ca_V_2.2-HA (solid blue circles, n = 10), Ca_V_2.2-HA E_I_A (solid red circles n = 8), Ca_V_2.2-HA E_IV_A (solid black circles, n = 6), and Ca_V_2.2-HA E_I, II, III, IV_A (solid green circles, n = 5) co-expressed with β1b and α_2_δ-1. For WT Ca_V_2.2-HA, the mean data were fit with a modified Boltzmann relationship (solid line). Fit data in Table S1. (D) I_Ba_ at +5 mV from the *I-V* relationships shown in (C). Individual data (same colors as C) and mean ± SEM are plotted. ^∗∗∗∗^p < 0.0001; ^∗∗∗^p = 0.0004 (one-way ANOVA with Dunnett’s multiple comparison correction compared to WT). (E) Representative whole-cell current traces from cells expressing WT Ca_V_2.2-HA (blue) or Ca_V_2.2-HA E_I, II, III, IV_A (green) with β1b and α_2_δ-1. Tail currents were recorded upon repolarization to −50 mV after test pulses between −50 and +70 mV from a holding potential of −100 mV. (F) Mean (±SEM) activation curves obtained from tail currents for WT Ca_V_2.2-HA (blue solid circles, n = 8) or Ca_V_2.2-HA E_I, II, III, IV_A (green solid circles, n = 12) were fitted with Boltzmann function. Fit data are in Table S1. All experiments in (B)–(F) used standard Cs Aspartate patch pipette solution. For all experiments, n = number of cells recorded from at least three separate transfections.

Here, we have examined the importance of the Ca_V_2 channel selectivity filter for channel trafficking, taking advantage of our previously described extracellular hemagglutinin (HA)-tagged Ca_V_2.2 construct, in which the tag does not affect function (Cassidy et al., 2014). Initially, we mutated the four pore-loop selectivity filter residues—E314, E664, E1370, and E1658—in domains I–IV of Ca_V_2.2 to the non-charged alanine (A), either alone or concurrently (Figure 1A). The Ca_V_2.2 E_I, II, III, IV_A selectivity filter mutant, in which all the mutations are present, is identical to the E4A selectivity filter mutant used by Cao and Tsien (2010), except for the extracellular HA tag to enable cell-surface expression to be determined (Cassidy et al., 2014). Importantly, in all our experiments, the α_1_ subunits were always expressed together with β1b and α_2_δ-1, unless otherwise stated.

Electrophysiological examination of these Ca_V_2.2 channels revealed that Ca_V_2.2 containing the substitution of E314 or E1658 to A (E_I_A or E_IV_A) retains a small inward Ba^2+^ current at +5 mV (Figures 1B–1D). More substantial mutations involving a combination of E to A pore mutations (E_II, IV_A; Figure S1), as well as a substitution of all pore glutamates (E_I, II, III, IV_A; Figures 1B–1D), showed no inward Ba^2+^ current at any potential. Furthermore, tail current measurements reinforced the absence of inward currents in Ca_V_2.2 E_I, II, III, IV_A (Figures 1E and 1F).

**Figure 2:**
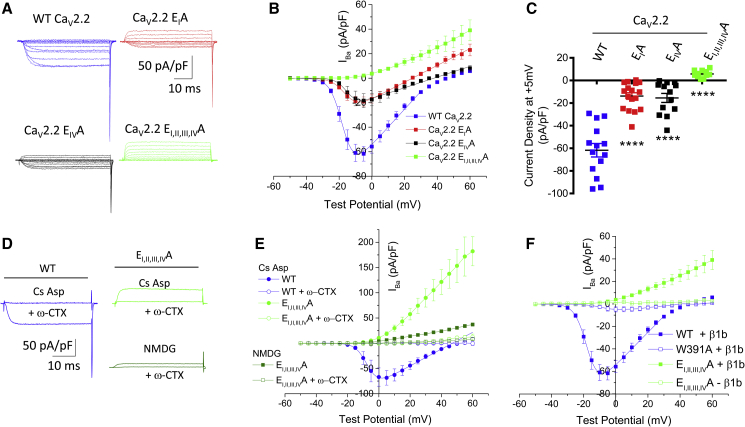
Calcium Channel Currents Produced by Selectivity Filter Mutant Ca_V_2.2 Channels in Comparison to WT Channels, Using an NMDG-Containing Pipette Solution (A) Example families of Ca_V_2.2 currents recorded from tsA-201 cells for WT Ca_V_2.2-HA (blue), Ca_V_2.2-HA E_I_A (red), Ca_V_2.2-HA E_IV_A (black), and Ca_V_2.2-HA E_I, II, III, IV_A (green), co-expressed with β1b and α_2_δ-1. Holding potential −80 mV, steps between −50 and +60 mV for 50 ms in 5-mV steps. (B) Mean (±SEM) *I-V* relationships for the conditions shown in (A). WT Ca_V_2.2-HA (solid blue squares, n = 14), Ca_V_2.2-HA E_I_A (solid red squares, n = 17), Ca_V_2.2-HA E_IV_A (solid black squares, n = 13), and Ca_V_2.2-HA E_I, II, III, IV_A (solid green squares, n = 11) co-expressed with β1b and α_2_δ-1. For all data except Ca_V_2.2-HA E_I, II, III, IV_A, the mean data were fit with a modified Boltzmann relationship, as shown (solid lines). (C) I_Ba_ at +5 mV from the *I-V* relationships shown in (B). Individual data (same colors as B) and mean ± SEM are plotted. ^∗∗∗∗^p < 0.0001 (one-way ANOVA with Dunnett’s multiple comparison correction compared to WT). (D) Examples of current traces at +5 mV of WT Ca_V_2.2-HA (left) or Ca_V_2.2-HA E_I, II, III, IV_A (right) recorded with either Cs Aspartate (top right) or NMDG (bottom right) in the internal solution before and after application of 1 μM ω-CTX. (E) Mean (±SEM) *I-V* relationships for conditions shown in (D). WT Ca_V_2.2-HA before (solid blue circles, n = 6) and after application of 1 μM ω-CTX (open blue circles, n = 6), Ca_V_2.2-HA E_I, II, III, IV_A before application of 1 μM ω-CTX recorded with either Cs Aspartate (solid light green circle, n = 9) or NMDG (solid dark green squares, n = 4), and after application of 1 μM ω-CTX recorded with either Cs Aspartate (open light green circles, n = 9) or NMDG (open dark green squares, n = 4). (F) Mean (±SEM) *I-V* relationships for WT Ca_V_2.2-HA + β1b (data repeated from panel B; solid blue squares, n = 14), Ca_V_2.2-HA W391A + β1b (open blue squares, n = 3), Ca_V_2.2-HA E_I, II, III, IV_A + β1b (data repeated from panel B; solid green squares, n = 11) and Ca_V_2.2-HA E_I, II, III, IV_ – β1b (open green squares, n = 3), all co-expressed with α_2_δ-1. For WT Ca_V_2.2-HA, the mean data were fit with a modified Boltzmann relationship, as shown (solid line). All experiments in (A)–(D) used NMDG-containing patch pipette solution. For all experiments, n = number of cells recorded from at least three separate transfections. Where possible, the mean data in (B), (E), and (F) were fit with a modified Boltzmann relationship (solid line). Fit data in Table S1.

Some of these Ca_V_2.2 mutants, including E_I_A and E_I, II, III, IV_A, exhibited a substantial outward current at +60 mV (Figures 1B, 1C, and S2A), likely because of the loss of selectivity of the pore, which becomes permeant to monovalent cations (Cs^+^ and K^+^) in the presence of a large driving force. In order to reduce outward currents through these channels, in case these might be masking residual inward current, we then utilized an N-methyl-D-glucamine (NMDG)-based internal solution (Figure 2). Indeed, replacing Cs Aspartate with NMDG significantly reduced the outward current observed, by about 80% at +60 mV (Figures 2A, 2B, and S2B), indicating it was carried mainly by Cs^+^. The results confirmed the absence of an inward current in Ca_V_2.2 E_I, II, III, IV_A and the presence of a small inward current in both the Ca_V_2.2 E_I_A and E_IV_A mutants (Figures 2A–2C).

We then performed several experiments that confirmed that the residual outward current seen in the Ca_V_2.2 E_I, II, III, IV_A mutant was through these channels. First, the Ca_V_2.2 E_I, II, III, IV_A outward current (recorded with either Cs Aspartate or NMDG internally), like the WT Ca_V_2.2 current, was lost when Ca_V_2.2 channels were blocked using ω-conotoxin GVIA (ω-CTX; Figures 2D and 2E).

Second, the outward current through the Ca_V_2.2 E_I, II, III, IV_A selectivity filter mutant channels was lost when the β subunit was omitted (Figure 2F). A similar result is obtained by the mutation of W391 to A in the AID in wild-type (WT) Ca_V_2.2 (Figure 2F) (Leroy et al., 2005).

### Trafficking of Selectivity Filter Mutant Ca_V_2.2 Channels Is Defective but Is Still Influenced by β Subunits

Although the presence of outward currents indicates some selectivity filter mutant channels are inserted in the plasma membrane, it is not possible to use this parameter for quantitative comparison because of the difference in ion conductance between the WT and selectivity filter mutant channels. We therefore examined the cell-surface expression of these channels, using the exofacial HA tag in Ca_V_2.2 to quantify channels in the plasma membrane in non-permeabilized Neuro2A (N2A) cells. In our initial experiments, we found that the selectivity filter mutant channels, co-expressed with α_2_δ-1 and β1b, were present in the plasma membrane to a much lower extent than WT Ca_V_2.2: a 53.5% reduction for Ca_V_2.2 E_I_A and a 67.8% reduction for Ca_V_2.2 E_I, II, III, IV_A (Figures S3A and S3B).

**Figure 3:**
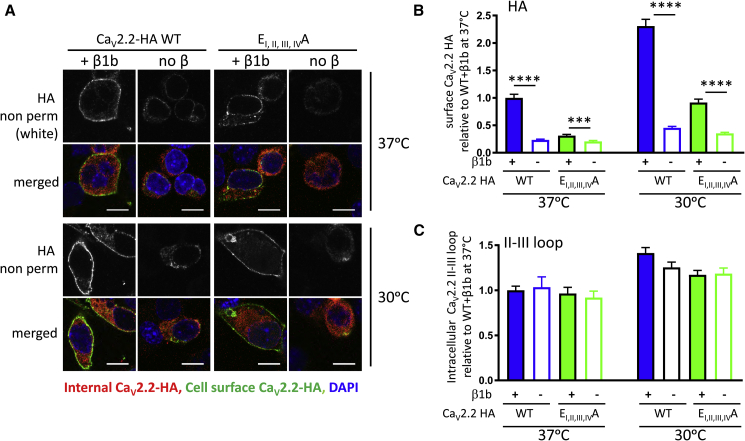
Effect of Mutations in the Ca_V_2.2 Selectivity Filter on Its Cell-Surface Expression and Stimulation by β subunit and by Reduced Temperature (A) Representative images of N2A cells expressing WT Ca_V_2.2-HA (left two columns) and Ca_V_2.2-HA E_I,II,III,IV_A (right two columns) with α_2_δ-1, in the presence (columns 1 and 3) and absence (columns 2 and 4) of β1b. Top row shows cell surface HA staining in non-permeabilized cells at 37°C for all conditions. Second row is the merged image for cells at 37°C; HA is shown in green, the Ca_V_2.2 II-III loop Ab staining after permeabilization is shown in red, and the nuclei are stained with DAPI (blue). The bottom two rows represent cells grown at 30°C. Scale bar, 10 μm. (B) Bar chart (mean ± SEM) for surface Ca_V_2.2-HA expression in N2A cells at 37°C (left four bars) and 30°C (right four bars) for WT Ca_V_2.2-HA with β1b (blue solid; n = 294 cells at 37°C, 306 cells at 30°C), WT Ca_V_2.2-HA without β1b (blue open; n = 146 cells at 37°C, 228 cells at 30°C), Ca_V_2.2-HA E_I, II, III, IV_A with β1b (green solid; n = 185 cells at 37°C, 251 cells at 30°C), and Ca_V_2.2-HA E_I, II, III, IV_A without β1b (green open; n = 130 cells at 37°C, 211 cells at 30°C). All conditions are with co-expression of α_2_δ-1. Data represent three independent experiments, which were normalized to the WT Ca_V_2.2-HA plus α_2_δ-1 and β1b at 37°C condition in each experiment. Statistical significance of the effect of the β subunit was determined using Student’s unpaired t test (^∗∗∗^p = 0.003; ^∗∗∗∗^p < 0.0001). The absence of β1b produced a 76.2% and 80.3% reduction for WT Ca_V_2.2-HA at 37°C and 30°C, respectively, and a 33.6% and 61.6% reduction for Ca_V_2.2-HA E_I, II, III, IV_A at 37°C and 30°C, respectively. (C) Bar chart (mean ± SEM) for staining with the intracellular Ca_V_2.2 II-III loop Ab after permeabilization. Conditions are those described for (B).

We then performed parallel experiments in the presence and absence of β subunits to determine whether the cell-surface expression of the selectivity filter mutant Ca_V_2.2 channels still responded to β-subunits, as we found to be the case in our electrophysiological experiments (see Figure 2F). These experiments were performed by comparing WT Ca_V_2.2 and Ca_V_2.2 E_I, II, III, IV_A, using HA Ab prior to permeabilization to determine cell-surface expression and a Ca_V_2.2 II–III loop Ab after permeabilization to determine intracellular Ca_V_2.2 expression (see Method Details). Cell-surface expression of Ca_V_2.2 E_I, II, III, IV_A was reduced by 68.9% compared to WT Ca_V_2.2 (Figures 3A and 3B), in agreement with the results above. While cell-surface expression of WT Ca_V_2.2 was reduced by 76.5% in the absence of β subunits, for Ca_V_2.2 E_I, II, III, IV_A, there was a smaller 33.5% reduction in the absence of β subunits (Figure 3B).

### Trafficking of Selectivity Filter Mutant Ca_V_2.2 Channels Remains Reduced at 30°C, a Temperature that Promotes Expression

Our finding that the cell-surface expression of the selectivity filter mutant channel Ca_V_2.2 E_I, II, III, IV_A appeared to be less affected than WT Ca_V_2.2 by the removal of the β subunit might be because the cell-surface level of the mutant channel is already extremely low and close to background levels. We therefore performed additional experiments with a period of cell growth at 30°C, a temperature at which expression and folding are promoted (Al-Fageeh et al., 2006). Indeed, both intracellular and cell-surface expression of Ca_V_2.2 were elevated after 30°C growth, relative to 37°C (Figures 3A–3C): for example, they were elevated by more than 2-fold for WT Ca_V_2.2 + β1b at the cell surface (Figure 3B). However, cell-surface expression of Ca_V_2.2 E_I, II, III, IV_A at 30°C was still reduced proportionately, relative to WT Ca_V_2.2—by 60.3%, a very similar reduction to that observed at 37°C. Furthermore, both the WT Ca_V_2.2 and Ca_V_2.2 E_I, II, III, IV_A still responded to the absence of a β subunit (an 80.3% reduction for WT Ca_V_2.2 and a 61.6% reduction for Ca_V_2.2 E_I, II, III, IV_A; Figures 3A and 3B). In contrast, intracellular Ca_V_2.2 was not consistently affected at both 30°C and 37°C by either of these manipulations (Figures 3A and 3C). Nevertheless, the effect of removing the β subunit was still less for the selectivity filter mutant channels, and this was reinforced in two additional sets of experiments, performed using different techniques to select transfected cells (Figures S3A–S3D), such that from four independent sets of experiments, the percent of reduction of cell-surface expression in the absence of β was 83.4% ± 4.5% for WT Ca_V_2.2 and 56.8% ± 8.8% for Ca_V_2.2 E_I, II, III, IV_A (p = 0.0174, paired t test). The fact that the selectivity filter mutant channels still responded significantly to both β-subunit co-expression and reduced temperature indicates that the Ca_V_2.2 E_I, II, III, IV_A channels are not grossly misfolded and thus aggregated in the ER. This is supported by the absence of increased co-localization of Ca_V_2.2 E_I, II, III, IV_A with an ER marker protein disulphide isomerase (Figure S3E).

### Function and Cell-Surface Expression of Selectivity Filter Charge Reversal Ca_V_2.2 E_IV_K Channels Are Severely Compromised

**Figure 4:**
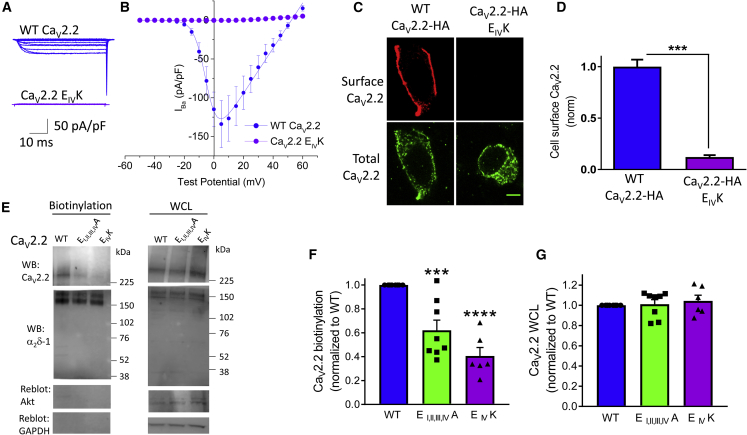
Effect of Charge Reversal Mutation E_IV_K in the Ca_V_2.2 Selectivity Filter on Its Function and Cell-Surface Expression (A) Current traces from tsA-201 cells expressing WT Ca_V_2.2-HA (blue) or Ca_V_2.2-HA E_IV_K (violet) together with β1b and α_2_δ-1 recorded with Cs Aspartate in the internal solution. Holding potential −80 mV, steps between −50 and +60 mV for 50 ms in 5-mV steps. (B) Mean (±SEM) *I-V* relationships for conditions shown in (A). WT Ca_V_2.2-HA (solid blue circles, repeated from Figure 1C) and Ca_V_2.2-HA E_IV_K (solid violet circles, n = 9). n = number of cells recorded from at least three separate transfections. (C) Example confocal images of WT (left) and E_IV_K (right) Ca_V_2.2-HA expressed in N2A cells. Cell-surface expression using HA Ab in non-permeabilized cells (top row); total expression using II-III loop Ab following permeabilization (bottom row). Scale bar, 5 μm. (D) Bar chart (mean ± SEM) for surface Ca_V_2.2 expression of WT Ca_V_2.2-HA/β1b/α_2_δ-1 (blue; n = 66 cells), in comparison to Ca_V_2.2-HA E_IV_K/β1b/α_2_δ-1 (violet; n = 70 cells). Statistical significance was determined using Student’s unpaired t test (^∗∗∗^p < 0.001). (E) Example immunoblot analysis of cell-surface biotinylated material (left panels) and WCL (right panels) for Ca_V_2.2-HA (top panel) WT (lane 1), E_I, II, III, IV_A (lane 2), and E_IV_K (lane 3), co-expressed with β1b and α_2_δ-1 (middle panel). Akt and GAPDH are loading and biotinylation controls (lower two panels). (F and G) Mean ± SEM (and individual data points, including those from data in E) for biotinylated (F) and WCL (G) Ca_V_2.2-HA WT (blue, n = 8), E_I, II, III, IV_A (green, n = 8), and E_IV_K (violet, n = 6) proteins, each normalized to control. In (F), ^∗∗∗^p = 0.0006 for Ca_V_2.2-HA E_I, II, III, IV_A and ^∗∗∗∗^p < 0.0001 for Ca_V_2.2-HA E_IV_K; one-way ANOVA with Dunnett’s post hoc multiple comparisons test, compared to WT.

One of the mutations causing episodic ataxia-2 (EA2) is a charge reversal mutation of the selectivity filter E in domain IV of Ca_V_2.1, which shows a severe reduction in function (Jeng et al., 2008). Indeed, we found that the same mutation in Ca_V_2.2 (Ca_V_2.2 E_IV_K) exhibited almost no inward or outward current (Figures 4A, 4B, and S1). In agreement with this, almost no cell-surface expression of the Ca_V_2.2 E_IV_K channel was observed (Figures 4C and 4D; see also Figures S4D and S4E). Cell-surface expression of WT Ca_V_2.2, Ca_V_2.2 E_I, II, III, IV_A, and Ca_V_2.2 E_IV_K (co-expressed with α_2_δ-1 and β1b) was also examined by cell-surface biotinylation in tsA-201 cells (Figures 4E and 4F). Similar results were obtained to those observed by immunocytochemistry in N2A cells, with a 38.0% reduction of plasma membrane expression measured by cell-surface biotinylation for Ca_V_2.2 E_I, II, III, IV_A and a 59.4% reduction for Ca_V_2.2 E_IV_K (Figures 4E and 4F). This experiment also showed that the total expression of these selectivity filter mutant channels, co-expressed with α_2_δ-1 and β1b, was not affected in the whole-cell lysate (WCL; Figure 4G).

### Trafficking of Selectivity Filter Mutant Ca_V_2.2 Channels Remains Sensitive to α_2_δ and Is Defective in Hippocampal Neurons

**Figure 5:**
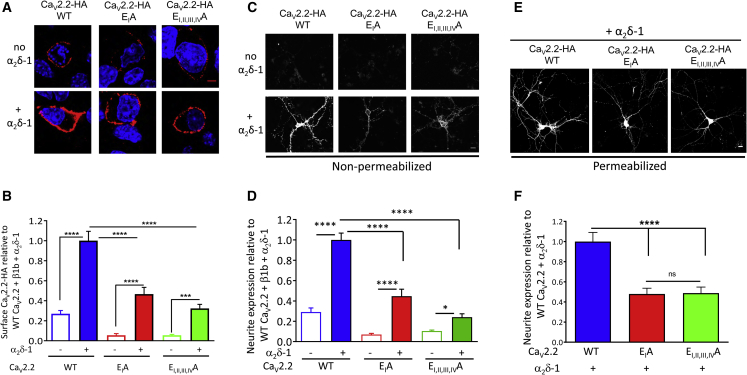
Effect of the Mutations in the Ca_V_2.2 Selectivity Filter on Its Cell-Surface Expression and Stimulation by α_2_δ-1 in N2A Cells and Hippocampal Neurites (A) Images of surface expression (HA Ab; red) of WT Ca_V_2.2-HA (left), Ca_V_2.2-HA E_I_A (middle), and Ca_V_2.2-HA E_I, II, III, IV_A (right) in non-permeabilized N2A cells, co-expressed with β1b alone (top panel) or with β1b + α_2_δ-1 (bottom panel). Nuclei stained with DAPI (blue). Scale bar, 5 μm. (B) Bar chart for surface Ca_V_2.2 expression of WT Ca_V_2.2-HA/β1b (blue open; n = 171 cells), WT Ca_V_2.2-HA/β1b/α_2_δ-1 (blue solid; n = 153 cells), Ca_V_2.2-HA E_I_A/β1b (red open; n = 101 cells), Ca_V_2.2-HA E_I_A/β1b/α_2_δ-1 (red solid; n = 112 cells), Ca_V_2.2-HA E_I,II,III,IV_A/β1b (green open; n = 153 cells), and Ca_V_2.2-HA E_I,II,III,IV_A/β1b/α_2_δ-1 (green solid; n = 234 cells) in N2A cells. Data are mean ± SEM normalized to the WT Ca_V_2.2-HA/β1b/α_2_δ-1 condition in each of four experiments. (C) Images of surface expression (HA Ab) for WT Ca_V_2.2-HA (left), Ca_V_2.2-HA E_I_A (middle), or Ca_V_2.2-HA E_I,II,III,IV_A (right) expressed in non-permeabilized hippocampal neurons when co-expressed with β1b alone (top panel) or with β1b + α_2_δ-1 (bottom panel). Scale bar of 20 μm applies to all images. (D) Bar charts for HA expression in neuronal processes of non-permeabilized hippocampal neurons transfected with WT Ca_V_2.2-HA/β1b (blue open; n = 124 processes, 33 cells), WT Ca_V_2.2-HA/β1b/α_2_δ-1 (blue solid; n = 140 processes, 41 cells), Ca_V_2.2-HA E_I_A/β1b (red open; n = 65 processes, 20 cells), Ca_V_2.2-HA E_I_A/β1b/α_2_δ-1 (red solid; n = 70 processes, 17 cells), Ca_V_2.2-HA E_I, II, III, IV_A/β1b (green open; n = 103 processes, 36 cells), or Ca_V_2.2-HA E_I, II, III, IV_A/β1b/α_2_δ-1 (green solid; n = 91 processes, 26 cells). Data are mean ± SEM neurite intensity/neuron. (E) Images of WT Ca_V_2.2-HA (left), Ca_V_2.2-HA E_I_A (middle), or Ca_V_2.2-HA E_I, II, III, IV_A (right) co-expressed with β1b and α_2_δ-1 and immunostained with the HA Ab in permeabilized hippocampal neurons. Scale bar of 20 μm applies to all images. (F) Bar charts for WT Ca_V_2.2-HA (blue; n = 242 processes, 72 cells), Ca_V_2.2-HA E_I_A (red; n = 220 processes, 60 cells), or Ca_V_2.2-HA E_I, II, III, IV_A (green; n = 175 processes, 50 cells), co-expressed with β1b and α_2_δ-1, in permeabilized hippocampal processes. Data are mean ± SEM neurite intensity/neuron. For (B), (D), and (F), statistical significance was determined using one-way ANOVA and Holm-Sidak’s multiple comparisons post hoc test (ns, non-significant; ^∗^p = 0.0467; ^∗∗∗^p < 0.0005; ^∗∗∗∗^p < 0.0001).

From a previous study (Kadurin et al., 2016), we found that trafficking of Ca_V_2.2 into hippocampal neurites is highly dependent on the presence of an α_2_δ subunit. We first examined the effect of α_2_δ-1 on trafficking of the Ca_V_2.2 selectivity filter mutants in undifferentiated N2A cells. Similar to previous results, cell-surface expression was reduced by 53.5% for Ca_V_2.2 E_I_A and by 67.8% for Ca_V_2.2 E_I, II, III, IV_A (Figures 5A and 5B). Furthermore, we found that the absence of the α_2_δ subunit reduced the channel density in the plasma membrane to a similar extent for the WT and mutant channels: by 73.1% for WT Ca_V_2.2, 83.0% for Ca_V_2.2 E_I_A, and 88.2% for Ca_V_2.2 E_I, II, III, IV_A (Figures 5A and 5B). Thus, binding of Ca^2+^ in the pore and/or conduction of Ca^2+^ are not prerequisites for the stimulation of Ca_V_2.2 trafficking by α_2_δ-1 in non-neuronal cells.

In order to examine the importance of an intact Ca^2+^ conduction pathway on calcium channel trafficking to the cell surface in neurons, we expressed the channels together with β1b and α_2_δ-1 in hippocampal neurons after 7 days in culture so that the neuronal processes were already extensive prior to transfection. In non-permeabilized neurons, we found a marked reduction in the density of Ca_V_2.2 expression in neurites for both selectivity filter mutant channels examined—by 76.3% for Ca_V_2.2 E_I_A and by 76.2% for Ca_V_2.2 E_I, II, III, IV_A—compared to WT Ca_V_2.2 (Figures 5C and 5D). This further highlights the importance of an intact selectivity filter for the trafficking of these channels. However, the absence of the α_2_δ subunit still decreased the penetration into neurites by the selectivity filter mutant channels to a similar extent to WT Ca_V_2.2: by 67.3% for WT Ca_V_2.2, 71.5% for Ca_V_2.2 E_I_A, and 56.3% for Ca_V_2.2 E_I, II, III, IV_A (Figure 5D).

To determine whether the trafficking defect exhibited by the selectivity filter mutants represents trafficking into the neurites themselves or insertion into the cell surface of the neurites, we examined the distribution of the channels in permeabilized neurons. We found total Ca_V_2.2 expression in the neurites was reduced compared to WT Ca_V_2.2 by 47.2% for Ca_V_2.2 E_I_A and by 53.6% for Ca_V_2.2 E_I, II, III, IV_A (all in the presence of β1b and α_2_δ-1; Figures 5E and 5F). Therefore, the main effect of the pore mutations in Ca_V_2.2 appears to be on the trafficking of these channels into the neurites, rather than specifically on cell-surface expression in the neurites.

### Trafficking of Selectivity Filter Mutant Ca_V_2.2 Channels into Presynaptic Terminals of Hippocampal Neurons Is Reduced

Ca_V_2 channels play a key role in neurotransmitter release, and their trafficking to presynaptic terminals is tightly controlled (for recent review, see Dittman and Ryan, 2019). We therefore examined the importance of an intact Ca^2+^ conduction pathway on calcium channel trafficking to the presynaptic terminals of hippocampal neurons in culture. For these experiments, we used N-terminally GFP-tagged Ca_V_2.2 constructs (Raghib et al., 2001) in addition to the extracellular HA tag. We did not find any evidence of cleavage of free GFP from these constructs (Figure S4A). Furthermore GFP-Ca_V_2.2 E_I, II, III, IV_A produced very similar outward currents to Ca_V_2.2 E_I, II, III, IV_A (Figures S4B and S4C) and showed a reduction in cell-surface expression by 86.9% compared to WT GFP-Ca_V_2.2; however, this remained sensitive to the removal of the β subunit (Figures S4D and S4E).

**Figure 6:**
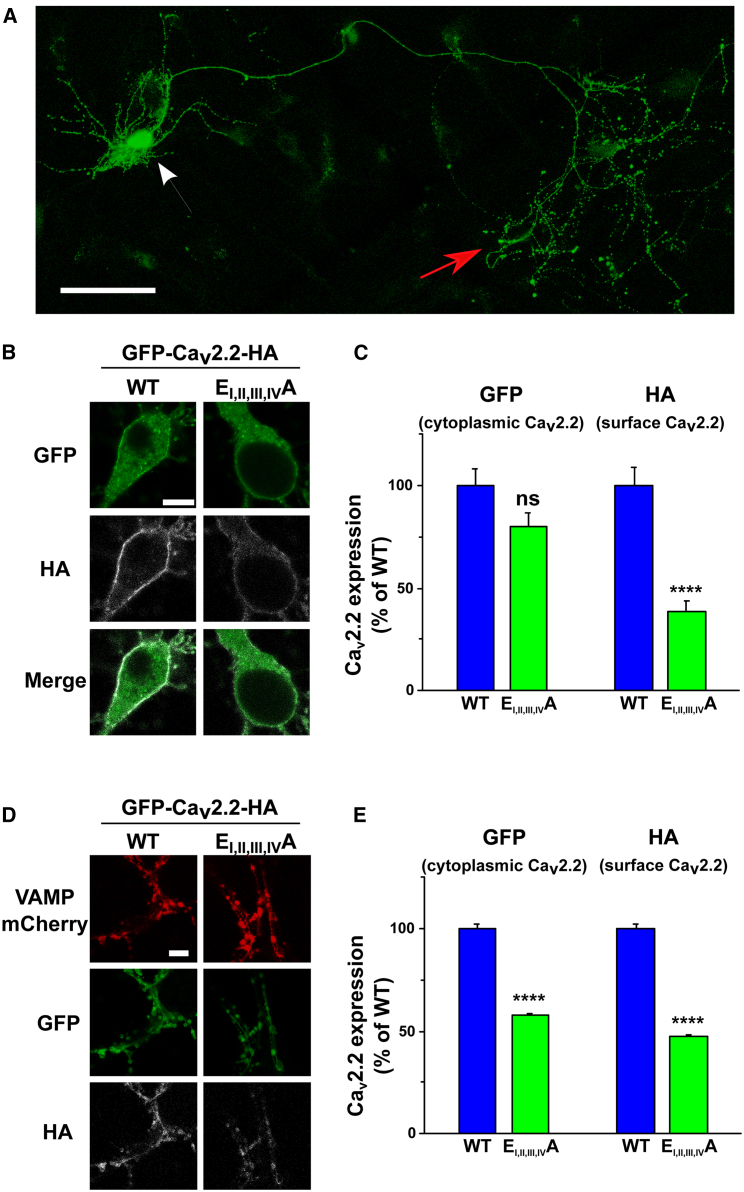
Effect of the Mutation in the Ca_V_2.2 Selectivity Filter on Its Expression in Hippocampal Neuronal Somata and Presynaptic Terminals (A) Confocal image of GFP fluorescence from a cultured hippocampal neuron expressing WT GFP-Ca_v_2.2-HA, α_2_δ-1, and β1b (2 × 2 tile, 20× objective and post-acquisition stitched). White arrow: soma, red arrow: presynaptic terminals. Scale bar, 100 μm. (B) Representative confocal images, in non-permeabilizing conditions, of soma expressing either WT (left panels) or E_I,II,III,IV_A mutant (right panels) GFP-Ca_v_2.2-HA, with α_2_δ-1 and β1b (63x objective). GFP (top panels), HA (white, middle panels), and merged images (bottom panels). Scale bar, 10 μm. (C) Bar chart (mean ± SEM) of GFP (cytoplasmic) and HA (cell surface) intensity in the soma. Data from 38 and 41 neurons for WT (blue bars) and E_I,II,III,IV_A mutant (green bars), respectively (from three independent experiments). Data were normalized to the WT in each experiment. ^∗∗∗∗^p < 0.0001. ns, p = 0.054. One-way ANOVA and Bonferroni post hoc test. (D) Confocal images, in non-permeabilizing conditions, of presynaptic terminals expressing WT (left panels) or E_I,II,III,IV_A mutant (right panels) GFP-Ca_v_2.2-HA with α_2_δ-1, β1b, and VAMP-mCherry (63× objective). VAMP-mCherry (red; top panels), GFP (green; middle panels), and HA (white; bottom panels). Scale bar, 10 μm. (E) Bar chart (mean ± SEM) showing GFP (cytoplasmic) and HA (cell surface) in synaptic terminals expressing GFP-Ca_V_2.2-HA. Data acquired from 1,280 and 1,873 boutons for WT (blue bars) and E_I,II,III,IV_A mutant (green bars), respectively (from between 22 and 25 synaptic areas from three independent experiments). Data were normalized to the WT in each experiment. ^∗∗∗∗^p < 0.0001. One-way ANOVA and Bonferroni post hoc test.

We therefore expressed in hippocampal neurons either WT or E_I, II, III, IV_A mutant GFP-Ca_V_2.2 constructs together with β1b and α_2_δ-1, with vesicle-associated membrane protein (VAMP)-mCherry as a marker for presynaptic terminals. Intracellular Ca_V_2.2 expression was monitored by the fluorescence of the GFP tag, and HA immunostaining in non-permeabilized conditions was used to assess its cell-surface expression (Figure 6).

In the somata of transfected neurons, the WT GFP-Ca_V_2.2 construct was strongly expressed on the cell surface, as detected by the HA-tag, and both at the plasma membrane and in the cytoplasm from the GFP fluorescence (Figures 6A–6C and S5). WT GFP-Ca_V_2.2 was also strongly expressed in the synaptic terminals of transfected neurons (Figures 6A, 6D, and 6E).

The GFP-Ca_V_2.2 E_I, II, III, IV_A mutant construct was expressed in the cytoplasm of the neuronal somata at a similar level to WT GFP-Ca_V_2.2 (Figures 6B and 6C); however, its expression on the somatic cell surface was reduced by 61% (Figures 6B and 6C). In contrast to WT GFP-Ca_V_2.2 (Figures S5A, S5C, and S5E), little juxta-membrane accumulation of GFP-Ca_V_2.2 E_I, II, III, IV_A was observed using GFP as a marker (Figures S5B, S5D, and S5F). However, no perinuclear accumulation of the mutant channel was observed (Figure S5B), which might have been indicative of a build-up of the newly translated channel in the ER (Waithe et al., 2011). At the level of presynaptic boutons, expression of the mutant channels was reduced by 42% in the cytoplasm and by 53% at the cell surface, in both cases compared to WT GFP-Ca_V_2.2 (Figures 6D and 6E). No HA immunostaining was seen in the absence of primary HA Ab (Figure S5G).

### Trafficking and Function of Ca_V_2.1 Selectivity Filter Mutant Channels Are Also Reduced Compared to WT Ca_V_2.1

**Figure 7:**
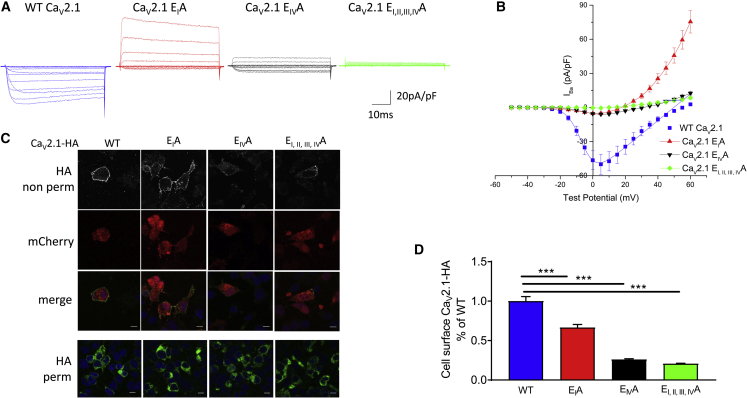
Effect of Mutations in the Ca_V_2.1 Selectivity Filter on Its Calcium Channel Currents and Cell Surface Expression (A) Example families of Ca_V_2.1 currents for WT Ca_V_2.1-HA (blue), Ca_V_2.1-HA E_I_A (red), Ca_V_2.1-HA E_IV_A (black), and Ca_V_2.1-HA E_I, II, III, IV_A (green), co-expressed with β1b and α_2_δ-1 in tsA-201 cells. Holding potential −80 mV, steps between −50 and +60 mV for 50 ms in 5-mV steps. Experiments used standard Cs Aspartate patch pipette solution. (B) Mean (±SEM) *I-V* relationships for the conditions shown in (A). WT Ca_V_2.1-HA (solid blue squares; n = 19), Ca_V_2.1-HA E_I_A (solid red squares; n = 10), Ca_V_2.1-HA E_IV_A (solid black squares; n = 12), and Ca_V_2.1-HA E_I, II, III, IV_A (solid green squares; n = 10) co-expressed with β1b and α_2_δ-1. For WT Ca_V_2.1-HA, the mean data were fit with a modified Boltzmann relationship (solid line). Fit data in Table S1. n = number of cells recorded from at least three separate transfections. (C) Representative images of cell-surface expression of Ca_V_2.1-HA (HA Ab; top row) in non-permeabilized N2A cells of (left to right) WT Ca_V_2.1-HA, Ca_V_2.1-HA E_I_A, Ca_V_2.1-HA E_IV_A, and Ca_V_2.1-HA E_I, II, III, IV_A. Second row shows mCherry transfection marker, and the third row shows merged images. Bottom row shows total expression of Ca_V_2.1-HA (HA Ab) in a separate experiment, in permeabilized N2A cells. All conditions contained β1b and α_2_δ-1. Scale bar, 10 μm. (D) Bar chart for surface Ca_V_2.1 expression in N2A cells of WT Ca_V_2.1-HA (blue bar; n = 429 cells), Ca_V_2.1-HA E_I_A (red bar; n = 360 cells), Ca_V_2.1-HA E_IV_A (black bar; n = 249 cells), and Ca_V_2.1-HA E_I, II, III, IV_A (green bar; n = 274 cells). All conditions contained β1b and α_2_δ-1. Data are mean ± SEM normalized to the WT Ca_V_2.1-HA condition. Statistical significance was determined using one-way ANOVA and multiple comparison post hoc test (^∗∗∗^p < 0.001). Data were obtained from at least three separate transfections.

We then made a Ca_V_2.1 channel construct with the HA tag at an equivalent site to that in Ca_V_2.2 (Ca_V_2.1-HA). This construct showed robust expression in the plasma membrane when co-expressed with β1b and α_2_δ-1 in tsA-201 cells, although only when using 30°C culture conditions (Figure S6A; see Method Details). It also supported substantial Ba^2+^ currents under the same conditions, although the current density for Ca_V_2.1-HA was reduced compared to WT Ca_V_2.1 without the HA tag (Figure S6B). We subsequently compared the properties of WT Ca_V_2.1-HA with Ca_V_2.1 E_I_A, E_IV_A and E_I, II, III, IV_A selectivity filter mutants. In contrast to WT Ca_V_2.1-HA, the Ca_V_2.1 E_I, II, III, IV_A selectivity filter mutant channel showed no inward current and only a small outward current (Figures 7A and 7B). For Ca_V_2.1 E_I_A and Ca_V_2.1 E_IV_A, the inward currents were very markedly reduced, although for Ca_V_2.1 E_I_A, a substantial outward current was present (Figures 7A and 7B).

We then examined the cell-surface expression of the Ca_V_2.1 selectivity filter mutants, and, in agreement with the electrophysiological results, we found that Ca_V_2.1 E_IV_A and Ca_V_2.1 E_I, II, III, IV_A showed almost no cell-surface expression in N2A cells (74.0% and 79.4% reduction, respectively), whereas clear cell-surface expression was observed for Ca_V_2.1-HA E_I_A, although it was 33.5% less than for WT Ca_V_2.1-HA (Figures 7C and 7D). In contrast, all constructs showed robust intracellular staining (Figure 7C, bottom row). Thus, the Ca_V_2.1 selectivity filter mutants also show very marked trafficking defects, which mirror closely the effects observed on the respective currents.

## Discussion

In this study, we set out to test the hypothesis that binding of Ca^2+^ in the selectivity filter of Ca_V_2 channels or conduction of Ca^2+^ by the channels may define an intracellular trafficking checkpoint for their optimal cell surface expression and represent a key stage in the assembly and trafficking of the channel complexes. We therefore examined whether Ca^2+^-impermeant Ca_V_2 channels, with mutations in their selectivity filters, were able to reach the plasma membrane in cell lines and neurons, and we found significant defects in their trafficking. Such selectivity filter mutant channels have been widely used in previous studies—initially to study permeation mechanisms (Ellinor et al., 1995, Sather et al., 1994, Yang et al., 1993) and more recently to show that Ca^2+^-dependent inactivation is mediated by Ca^2+^ binding to residues near the selectivity filter of Ca_V_1.2 (Abderemane-Ali et al., 2019). However, they have also been used in physiological experiments as non-functional “dominant negative” channels (Cao et al., 2004, Cao and Tsien, 2010, Krey et al., 2013) or as channels that do not conduct Ca^2+^ (Servili et al., 2018).

By examining in parallel the expression and trafficking of several selectivity filter mutant channels, both in cell lines and in hippocampal neurons, we obtained the unexpected result that Ca_V_2.2 selectivity filter mutant channels have a severely compromised ability to traffic to the cell surface, despite being co-expressed with the relevant α_2_δ and β auxiliary subunits. We found very similar results for Ca_V_2.1 selectivity filter mutants, except that trafficking of Ca_V_2.1 E_I, II, III, IV_A was even more severely compromised than for the equivalent Ca_V_2.2 mutant, suggesting that trafficking of this channel is very sensitive to interference with the structural integrity of the selectivity filter. It is of great interest that two *CACNA1A* EA2 missense mutations have been identified that convert the selectivity filter glutamate in domain IV either to the oppositely charged lysine (Denier et al., 2001) or to glycine (Petrovicova et al., 2017). We found that the Ca_V_2.1 E_IV_A mutant (Figure 7D) and the Ca_V_2.2 E_IV_K mutant (Figure 4) exhibited very little surface expression.

In our experiments, all Ca_V_ α_1_ constructions were initially co-expressed with auxiliary subunits. The β subunits are extremely important for both the correct function of Ca_V_1 and 2 channels (Pragnell et al., 1994) and their trafficking to the plasma membrane and into neuronal processes (Altier et al., 2011, Cassidy et al., 2014, Waithe et al., 2011). The β subunits promote α-helix formation in the I-II linker to which they bind (Opatowsky et al., 2004, Van Petegem et al., 2008) and also reduce polyubiquitination and ER-associated proteasomal degradation of the channels (Altier et al., 2011, Page et al., 2016, Waithe et al., 2011). Here, we show that the trafficking of the selectivity filter mutant Ca_V_2.2 channels still responded to β subunits, albeit to a lesser extent than WT channels, suggesting that Ca^2+^ binding in the channel selectivity filter may represent an early step in channel folding in the ER, priming the channel to respond optimally to β-subunit binding. Furthermore, the defect in the trafficking of selectivity filter mutant Ca_V_2.2 channels cannot be overcome by cell growth at a reduced temperature, which promotes correct protein folding. While it is an intriguing possibility, it is not possible to determine conclusively from this study whether it is indeed the absence of Ca^2+^ binding in the selectivity filter that mediates this trafficking defect, since the selectivity filter mutations themselves or a combination of both issues may be responsible. However, our results indicate that the reduced trafficking of the selectivity filter mutant channels is not because they are so misfolded that they are aggregated within the ER, which might render their trafficking completely insensitive to β-subunit binding and result in proteasomal degradation. This is not surprising, since interference with the selectivity filter by side chain substitution does not alter the carbonyl backbone and should not cause a complete collapse of the pore.

We have shown previously that α_2_δ subunits are also important for the optimal trafficking of Ca_V_2 channels to the plasma membrane (Cassidy et al., 2014), and the binding of the α_2_δ VWA domain to the first extracellular loop in domain I is essential for this effect (Dahimene et al., 2018). In a recent study using non-cleavable α_2_δ subunits, which were unable to increase channel function, we found major trafficking defects of the channel complex in neurons (Ferron et al., 2018, Kadurin et al., 2016). Moreover, the presence of an α_2_δ subunit was required to observe enhanced trafficking resulting from trafficking motifs in exon 37a in the proximal C terminus of Ca_V_2.2 (Macabuag and Dolphin, 2015). However, here we found that trafficking of the divalent cation-impermeant selectivity filter mutant Ca_V_2.2 channels still responded proportionately to α_2_δ subunits, indicating that a lack of interaction with α_2_δ was not the reason for the reduced cell-surface expression of the impermeant channels.

Our results indicate that binding of Ca^2+^ in the pore selectivity filter may be required for optimal trafficking of the Ca_V_2 channels, and although this result is obtained following heterologous expression, it is likely to represent a valid conclusion. It has been shown that Ca^2+^ is bound tightly in the pore of WT L-type Ca^2+^ channels, even when extracellular Ca^2+^ is reduced to μM levels, such that there is no permeation (Yang et al., 1993). It is therefore possible that the binding of Ca^2+^ ions to the Ca_V_2 channel selectivity filter, which could take place as the channel folds in the high Ca^2+^ environment of the ER, plays a role in optimizing channel structure. This effect of Ca^2+^ binding might occur in synergy with the effect of β-subunit binding to the AID in the I-II linker. The β-subunit interaction has been shown to promote α-helix formation in the AID that extends into transmembrane segment IS6 (for review, see Minor and Findeisen, 2010). The formation of a mature structure of the core of the Ca_V_ channel pore domain may then promote the exposure of particular intracellular juxta-membrane trafficking motifs, such as those binding adaptor proteins (Macabuag and Dolphin, 2015), which facilitate trafficking beyond the ER. The interaction of Ca_V_ channels, via other important trafficking motifs in the C terminus and elsewhere, with their targets in neurons might also be affected (Kaeser et al., 2011, Maximov and Bezprozvanny, 2002, Tseng et al., 2017). A similar process has been identified for ionotropic glutamate receptors, in which mutations of the glutamate agonist binding site prevent exit from the ER, leading to the suggestion that agonist binding in the ER is a part of a quality control checkpoint for channel maturation and trafficking (Daou et al., 2013). Alternatively, it may be that Ca_V_ channels that are rendered non-functional or poorly functional by other means will show reduced trafficking, and this will require future experimentation.

A corollary of our results is that physiological experiments, in which selectivity filter mutants of Ca_V_2 channels have been used as dominant-negative or Ca^2+^-impermeant channels (Cao et al., 2004, Cao and Tsien, 2010, Krey et al., 2013), may need reinterpretation. Such Ca_V_2.1 and Ca_V_2.2 mutants have been used previously in the elaboration of the “slots” hypothesis, whereby a limited number of Ca_V_2.1-preferring slots would be associated with each vesicular release site in presynaptic terminals, and these could be occupied by either WT channels or experimentally by selectivity filter mutant non-permeant channels (Cao et al., 2004). However, in the present study, we show that the assumption that the selectivity filter mutants would have the same trafficking properties as their WT counterparts is incorrect; in fact, they exhibit severe trafficking defects. In addition, a more recent study (Lubbert et al., 2019) in the giant Calyx of Held synapse, which is optimized for fidelity of transmission (Borst and Sakmann, 1998, Mc Laughlin et al., 2008), provides no evidence for limiting Ca_V_2.1-preferring slots.

Furthermore, in these and other previous studies, the channels were overexpressed in the absence of auxiliary subunits, whereas our work has highlighted the importance of both β and α_2_δ in trafficking calcium channels into neuronal processes (Kadurin et al., 2016, Waithe et al., 2011) and into presynaptic active zones to support vesicular release by endogenous Ca^2+^ channels (Ferron et al., 2018, Hoppa et al., 2012). Our work shows it is inadvisable to overexpress either WT or mutant calcium channel α_1_ subunits in neurons without additional β and α_2_δ subunits, on the assumption that these auxiliary subunits would be present endogenously in great excess, as this is unlikely to be the case. If channels are expressed at the same low level as present endogenously, then this will not be an issue.

The skeletal muscle Ca_V_1.1 channel does not rely on Ca^2+^ permeation for its main function, and it is of interest that a previous study showed that the expression of a selectivity filter mutant in Ca_V_1.1 (E_III_K) remained able to restore both off-gating currents and excitation-contraction coupling when expressed in dysgenic myotubes, whereas the equivalent Ca_V_1.2 mutant did not (Dirksen and Beam, 1999). Therefore, trafficking of Ca_V_1.1 into their stable tetrad organization within t-tubule membranes may be less dependent on Ca^2+^ binding in its selectivity filter; indeed, the trafficking of these channels is also independent of α_2_δ-1 (Gach et al., 2008). Other recent studies, which have used Ca_V_1.2 channels with mutations in or adjacent to the selectivity filter, have made several important findings. First, the conserved aspartate adjacent to the selectivity filter E in domain II is an essential mediator of Ca^2+^-dependent inactivation (Abderemane-Ali et al., 2019); and second, Ca^2+^ occupation of the selectivity filter, but not permeation, was found to be essential to mediate effects of Ras (Servili et al., 2018). Whether mutation of the selectivity filter in L-type channels affects their cell-surface expression remains to be determined, the result of which might alter the interpretation of these experiments.

### Conclusions

Our results show a very marked trafficking defect in selectivity filter mutant Ca_V_2 channels and further suggest the possibility that a combination of interaction of Ca_V_2.2 channels with the auxiliary subunits, together with Ca^2+^ binding in the pore, may promote correct folding and trafficking of the channels.

## STAR★Methods

### Key Resources Table

**Table:** 

REAGENT or RESOURCE	SOURCE	IDENTIFIER
**Antibodies**		

Anti-Ca_V_2.2 II-III loop Ab (rabbit polyclonal)	Raghib et al., 2001	n/a
Anti-Ca_V_2.1 Ab	Alomone labs	Cat #ACC-001; RRID: AB_2039764
Anti-HA ab rat monoclonal	Sigma-Aldrich	Clone 3F10, Cat #11815016001; RRID: AB_390914
Anti-HA Ab rabbit polyclonal	Sigma-Aldrich	Cat #H6908; RRID: AB_260070
Anti-PDI Ab mouse monoclonal	Abcam	Cat #ab2792; RRID: AB_303304
Anti-GAPDH ab	Ambion	Cat #AM4300; RRID: AB_2536381
Anti-Akt Ab (rabbit polyclonal)	Cell Signaling Technology	Cat #9272; RRID: AB_329827
Anti-rabbit Alexa fluor 594	Thermo Fisher	Cat #R37117; RRID: AB_2556545
Anti-rat Alexa fluor 488	Thermo Fisher	Cat #A-11006; RRID: AB_2534074
Anti-mouse Alexa fluor 647	Thermo Fisher	Cat #A32728; RRID: AB_2633277
Anti-mouse Alexa fluor 488	Thermo Fisher	Cat #A-11001; RRID: AB_2534069
Anti-rat Alexa fluor 594	Thermo Fisher	Cat #A-11007; RRID: AB_10561522
Anti-rat fluorescein isothiocyanate	Sigma-Aldrich	Cat #F1763; RRID: AB_259443
Goat anti-rabbit HRP	Biorad, Hemel Hempstead, UK	Cat #1706515; RRID: AB_11125142
Goat anti-rat HRP	Biorad, Hemel Hempstead, UK	Cat #5204-2504; RRID: AB_619913
Goat anti-mouse HRP	Biorad, Hemel Hempstead, UK	Cat #1721011; RRID: AB_11125936
Anti-Rat IgG Biotin antibody	Sigma-Aldrich	Cat #B7139; RRID: AB_258601
Streptavidin Alexa Fluor 594	Thermo Fisher	Cat #S11227

**Chemicals, Peptides, and Recombinant Proteins**		

ω-conotoxin GVIA	Alomone	Cat #C-300
Penicillin-Streptomycin (10,000 U/mL)	Invitrogen	Cat #15140-122
Poly-L-lysine	Sigma	Cat #P.6282
Dulbecco’s modified Eagle’s medium	Thermo Fisher	Cat #41965-039
GlutaMAX	Invitrogen	Cat #35050-038
Fugene	Promega	Cat #E2311
Polyjet	Tebu-bio Ltd	Cat #189-SL100688-1
Opti-MEM	Thermo Fisher	Cat #41965-039
Neurobasal Medium	Invitrogen	Cat #10888-022
B27 medium	Thermo Fisher	Cat #17504044
HEPES	Sigma	Cat #H3375
Horse serum	Invitrogen	Cat #26050-088
Lipofectamine 2000	Invitrogen	Cat #L3000-008
Premium Grade EZ-link Sulfo-NHS-LC-Biotin	Thermo Scientific	Cat #21335
Glycine	Sigma	Cat #G8898
Sodium Dodecyl Sulfate	VWR	Cat #444062F
Protease Inhibitors	Roche	Cat #11697498001
Dithiothreitol	Melford	Cat #MB1015
3–8% Tris-Acetate gels	Invitrogen	Cat #EA0375BOX
polyvinylidene fluoride (PVDF) membrane	Biorad	Cat #1620177
streptavidin-agarose beads	Thermo Scientific	Cat #20347
Igepal	Sigma	Cat #I3021
A/G PLUS Agarose slurry	Santa Cruz	Cat #Sc-2003
Paraformaldehyde	sigma	Cat #P6148
Goat serum	Invitrogen	Cat #6210-072
Triton X-100	Thermo Scientific	Cat #28314
4’,6-diamidine-2′-phenylindole dihydrochloride (DAPI)	Molecular probes	Cat #nl5995050
VectaShield Vector Laboratories	Vector Laboratories	Cat #H1000
fetal bovine serum	Invitrogen	Cat #10270
Bovine serum albumin	First Link (UK) Ltd	Cat #41-00-410
Papain	Sigma	Cat # P4762
L-Cysteine	Sigma	Cat #W326305
DNase I	Invitrogen	Cat #18047019
Glucose	Sigma	Cat #G7528
Hanks buffered salt solution (HBSS)	Invitrogen	Cat #14175053

**Critical Commercial Assays**		

Bradford Assay	Biorad	Cat #500-0006
ECL 2	Thermo scientific	Cat #32132

**Experimental Models: Cell Lines**		

tsA-201 cells	ECACC	Cat #96121229; RRID: CVCL_2737
Neuro2A cells	ATCC	Cat #CCL-131; RRID: CVCL_0470

**Experimental Models: Organisms/Strains**		

Rat Sprague Dawley male	UCL, bred in house	n/a

**Oligonucleotides**		

Table S2	This paper	n/a

**Recombinant DNA**		

Rabbit Ca_V_2.2-HA	Cassidy et al., 2014	n/a
Ca_V_2.2-HA E_I_A	This paper	n/a
Ca_V_2.2-HA E_IV_A	This paper	n/a
Ca_V_2.2-HA E_I, II, III, IV_A	This paper	n/a
Ca_V_2.2-HA E_IV_K	This paper	n/a
Rat β1b (GenBank: X61394)	Pragnell et al., 1991	RRID: Addgene_107423
β1b-GFP	Waithe et al., 2011	RRID: Addgene_89893
Rat α_2_δ-1 (GenBank: M86621)	Kim et al., 1992	RRID: Addgene_58726
Rat Ca_V_2.1 (GenBank: M64373)	Brodbeck et al., 2002	n/a
Ca_V_2.1-HA	This paper	n/a
Ca_V_2.1-HA E_I_A	This paper	n/a
Ca_V_2.1-HA E_IV_A	This paper	n/a
Ca_V_2.1 HA E_I, II, III, IV_A	This paper	n/a
GFP-Ca_V_2.2 HA	Macabuag and Dolphin, 2015	n/a
GFP-Ca_V_2.2-HA E_I, II, III, IV_A	This paper	n/a
mcherry (GenBank: AY678264)	Shaner et al., 2004	n/a
CD8	Rougier et al., 2005	n/a
Vamp-mCherry	Hoppa et al., 2012	n/a

**Software and Algorithms**		

ImageJ	NIH	https://imagej.nih.gov/ij/RRID:SCR_003070
GraphPad Prism 5 or 7	GraphPad software	https://www.graphpad.com/scientific-software/prism/
Origin-Pro 2015	Microcal Origin, Northampton, MA	https://www.originlab.com/origin
pCLAMP 9	Molecular Devices	https://www.moleculardevices.com/products/axon-patch-clamp-system/acquisition-and-analysis-software/pclamp-software-suite

### Lead Contact and Materials Availability

Further information and requests for resources and reagents should be directed to and will be fulfilled by the Lead Contact, Annette Dolphin (a.dolphin@ucl.ac.uk).

#### Materials availability statement

Addgene catalog numbers are given in Key Resources table and additional constructs will be deposited in Addgene.

### Experimental Model Details

#### Cell lines

The cell lines were plated onto cell culture flasks, coverslips or glass-bottomed dishes (MatTek Corporation, Ashland, MA), coated with poly-L-lysine, and cultured in a 5% CO_2_ incubator at 37°C.

#### tsA-201 cell line

The tsA-201 cells (European Collection of Cell Cultures, female sex) were cultured in Dulbecco’s modified Eagle’s medium (DMEM) supplemented with 10% fetal bovine serum (FBS), 1 unit/ml penicillin, 1 μg/ml streptomycin and 1% GlutaMAX (Life Technologies, Waltham, MA).

#### N2A cell line

Mouse neuroblastoma N2A cells (American Tissue culture collection # CCL-131, male sex) were obtained from Professor Roger Morris, Kings College London (Parkyn et al., 2008). They were cultured in 50% DMEM and 50% OPTI-MEM supplemented with 5% FBS, 1 unit/ml penicillin, 1 μg/ml streptomycin, and 1% GlutaMAX.

#### Primary rat hippocampal cultures

All experiments were performed using a Schedule 1 method in accordance with the Home Office Animals (Scientific Procedures) Act 1986, UK. Hippocampal neurons were obtained from male P0 Sprague Dawley rat pups. After cervical dislocation and decapitation, brains were excised and rapidly placed in ice-cold Hanks buffered salt solution (HBSS, Life Technologies) and hemisected. After dissecting out the mid brain from each hemisphere, hippocampi were collected and cleaned from the meninges. Hippocampi were chopped into small pieces and incubated in a shaker (200 rpm) for 40 min at 37°C in a HBSS-based dissociation solution containing: papain (Sigma, 7 unit/ml), bovine serum albumin (BSA, 0.2 mg/ml), L-Cysteine (0.2 mg/ml), glucose (5 mg/ml) and DNase I (Life Technologies, 1000 unit/ml). The digested tissues were then gently triturated with glass Pasteur pipettes and the cells were counted. Approximately 75 × 10^3^ cells in 100 μL of plating solution (Neurobasal medium supplemented with B27 (Life Technologies; 2%), HEPES (10 mM), horse serum (5%), glutamine (0.5 mM), and 1 unit/ml penicillin, 1 μg/ml streptomycin) were seeded onto sterile poly-lysine-coated glass coverslips and incubated at 37°C. After 2 h, the plating solution was replaced with 1 mL of growth medium (serum-free Neurobasal medium supplemented with B27 (Life Technologies; 4%), 2-mercaptoethanol (25 μM), glutamine (0.5 mM), and 1 unit/ml penicillin, 1 μg/ml streptomycin), half of which was replaced every 3-4 days.

### Method Details

#### Molecular biology

The following α_1_-subunit cDNAs were used: Ca_V_2.2 (rabbit, GenBank: D14157), always containing an extracellular HA-tag (Cassidy et al., 2014), with an additional N-terminal GFP tag where stated (Macabuag and Dolphin, 2015, Raghib et al., 2001) and rat Ca_v_2.1 (GenBank: M64373 with E1686R) (Brodbeck et al., 2002), with an HA-tag in an equivalent position, except when stated. For this construct, two HA sequences separated by a Gly residue were inserted between V572 and I573 to give [.....VIWAV-(HA)-G-(HA)-IKPGT...]. Site-directed mutagenesis to make selectivity filter mutant constructs of Ca_V_2.2 and Ca_V_2.1 was carried out using standard procedures, and all subcloning and mutations were confirmed by sequencing. Oligonucleotides used to make the new constructs used in this study are given in Table S2. Other cDNAs used were β1b (rat, GenBank: X61394) (Pragnell et al., 1991), β1b-GFP (Waithe et al., 2011), α_2_δ-1 (rat, GenBank: M86621) (Kim et al., 1992), mCherry (Shaner et al., 2004) and VAMP-mCherry (Hoppa et al., 2012). The cDNAs were in the pcDNA3 vector for expression in tsA-201 and N2A cells and pcDNA3 or pCAGGS for expression in hippocampal neurons. CD8 cDNA (Rougier et al., 2005) was included as a transfection marker where stated.

#### Antibodies and other materials

Antibodies (Abs) used were: anti-Ca_V_2.2 II-III loop Ab (rabbit polyclonal) (Raghib et al., 2001), anti-Ca_V_2.1 II-III loop Ab (rabbit polyclonal, Alomone), anti- α_2_δ-1 Ab (mouse monoclonal, Sigma-Aldrich), anti-HA (rat monoclonal, Roche), anti-HA (rabbit polyclonal, Sigma), anti-PDI (mouse monoclonal, Abcam), anti-Akt Ab (rabbit polyclonal, Cell Signaling Technology) and anti-GAPDH Ab (mouse monoclonal, Ambion). For immunocytochemistry, secondary Abs (1:500) used were anti-rabbit-Alexa Fluor 594, anti-rat- Alexa Fluor 488, anti-rat-Alexa Fluor 594 and 647 and anti-mouse-Alexa Fluor 488 (ThermoFisher). For immunoblotting, secondary Abs (1:2000) were anti-rabbit-Horseradish Peroxidase (HRP), and anti-mouse HRP (Biorad).

#### Cell line transfection

For immunocytochemistry, tsA-201 cells and N2A cells were transfected with Ca_V_2.1-HA or Ca_V_2.2-HA together with α_2_δ-1 and β1b (unless otherwise stated, all in vector pcDNA3) in a ratio 3:2:2 (plus 0.5 for mCherry as a transfection marker when included). The transfection reagent used was PolyJet (Tebu-bio Ltd), used in a ratio of 3:1 to DNA mix, according to the manufacturer’s protocol. Culture medium was changed 6 h after transfection and cells were either incubated at 37°C for a further 42 h or transferred to 30°C for 66 h. For electrophysiological studies, tsA-201 cells were transfected as above, except using Fugene6 (Promega, Fitchburg, WI), according to the manufacturer’s protocol. β1b-GFP or CD8 were used as transfection markers. Similar data were obtained using the two different methods to identify transfected cells for recording (CD8 in all Figures, except Figure S1, which used β1b-GFP). Cells were incubated at 37°C post transfection or incubated at 37°C overnight and then transferred to 30°C for a further 24 h, where stated. For cell surface biotinylation experiments, tsA-201 cells were transfected as above using Fugene6 and incubated at 37°C for 48 h.

#### Neuronal transfection

At 7 days *in vitro* and 2 h before transfection, half of the medium was removed, and kept as ‘conditioned’ medium, and 500 μL of fresh medium was added. The hippocampal cultures were then transfected using Lipofectamine 2000, at a ratio of 1:2 to DNA mix (1 μg/μl). After 2 h, the transfection mixes were replaced with growth medium consisting of 50% conditioned and 50% fresh medium. The DNA mix (in pCAGGS) consisted of Ca_V_2.2 (either WT-Ca_V_2.2-HA, GFP-Ca_V_2.2 HA, Ca_V_2.2-HA E_I_A, Ca_V_2.2-HA E_I,II,III,IV_A or GFP-Ca_V_2.2-HA E_I,II,III,IV_A), α_2_δ-1, β1b and mCherry or VAMP-mCherry, at a ratio of 3:2:2:1. α_2_δ-1 was replaced by empty vector when appropriate. The cultures were used for immunostaining experiments at 14 days *in vitro*, as described below.

#### Cell surface biotinylation and immunoblotting

Cell surface biotinylation experiments were carried out on tsA-201 cells expressing either WT Ca_V_2.2-HA or the E_I, II, III, IV_A and E_IV_K pore mutants together with α_2_δ-1 and β1b. At 48 h after transfection, cells were rinsed with phosphate-buffered saline (PBS) and then incubated for 30 min at room temperature (RT) with 0.5 mg/ml Premium Grade EZ-link Sulfo-NHS-LC-Biotin (Thermo Scientific) in PBS. The reaction was quenched by removing the biotin solution and replacing with PBS containing 200 mM glycine for 2 min at RT. The cells were rinsed with PBS before being resuspended in PBS containing 1% Igepal; 0.1% SDS and protease inhibitors (PI, cOmplete, Roche), pH 7.4, for 30 min on ice to allow cell lysis, cleared by centrifugation at 13,000 × g and assayed for total protein (Bradford assay, Biorad). Cleared WCLs corresponding to 20 - 40 μg total protein were mixed with 5 x Laemmli sample buffer to a final dilution of 1 x (Davies et al., 2010), and were supplemented with dithiothreitol (DTT) to a final concentration of 100 mM. The samples were then resolved by SDS-polyacrylamide gel electrophoresis (PAGE) on 3–8% Tris-Acetate gels (Invitrogen) and transferred to polyvinylidene fluoride (PVDF) membrane (Biorad). The membranes were blocked with 5% BSA, 0.5% Igepal in Tris-buffered saline (TBS) for 30 min at RT and then incubated overnight at 4°C with the relevant primary Ab. After washing in TBS containing 0.5% Igepal, membranes were incubated with the appropriate secondary Ab for 1 h at RT. The signal was obtained by HRP reaction with fluorescent product (ECL 2; Thermo Scientific) and membranes were scanned on a Typhoon 9410 phosphorimager (GE Healthcare). Biotinylated lysates (equalized to between 0.5 and 1 mg/ml total protein concentration) were applied to 40 μl prewashed streptavidin-agarose beads (Thermo Scientific) and rotated overnight at 4°C. The streptavidin beads were then washed 3 times with PBS containing 0.1% Igepal, resuspended in an equal volume of 2 x Laemmli buffer, supplemented with DTT to a final concentration of 100 mM, and heated for 10 min at 56°C to elute the precipitated protein. The samples containing cell surface proteins were then analyzed by immunoblotting with the indicated Abs as described above.

#### Immunocytochemistry, imaging and analysis

Immunocytochemistry was carried out on N2A cells expressing either Ca_V_2.1-HA or Ca_V_2.2-HA (WT or selectivity filter mutants) together with α_2_δ-1 and β1b. After transfection, cells were incubated at either 30°C or 37°C, as described, for 48-72 h before being fixed with 4% paraformaldehyde (PFA) in PBS, pH7.4 at RT for 5 min. When permeabilization was required, cells were then incubated in PBS containing 0.2% Triton X-100 for 5 min at RT. Blocking was performed for 30 min at RT in PBS containing 20% goat serum and 5% BSA. For staining of extracellular epitopes, blocking, and primary Ab incubation steps were carried out prior to permeabilization. The indicated primary Abs were applied (diluted in PBS with 10% goat serum and 2.5% BSA) overnight at 4°C or for 1 h at RT. The indicated secondary Abs were applied (1:500 dilution in PBS, containing 2.5% BSA and 10% goat serum) at RT for 1 h. Cell nuclei were stained with 0.5 μM 4’,6’-diamidino-2-phenylindole (DAPI) in PBS for 5 min. For surface/intracellular HA staining of Ca_V_2.2-HA as shown in Figure S3, cells were incubated with 1:500 anti-HA (rat, monoclonal) for 1 h, followed by incubation with 1:500 anti-rat biotin 1h - effectively masking surface HA epitopes -, permeabilization as described above, followed by incubation with 1:500 anti-HA (rat, monoclonal) overnight at 4°C to probe for intracellular HA epitopes, and finally incubation with 1:500 Steptavidin 594 and anti-rat Alexa Fluor 488.

Cultures of transfected hippocampal neurons were fixed, after 14 days *in vitro*, in PBS containing 4% PFA / 4% sucrose for 5 min at RT, and then blocked in PBS containing 20% goat serum and 5% BSA for 30 min at RT. Primary Ab (rat anti-HA, 1:200 dilution) was applied overnight at 4°C. Coverslips were washed and cells were then permeabilized by incubating with 0.2% Triton X-100 in PBS for 5 min at RT. When required, the second primary Ab (anti-Ca_V_2.2 II-III loop, 1:250) was incubated overnight at 4°C. The appropriate secondary Abs were applied for 1 h at RT. The coverslips were mounted onto glass slides using VECTASHIELD® mounting medium (Vector Laboratories, Peterborough, UK).

Imaging was performed on Zeiss LSM 780 confocal microscope, at fixed microscope settings for all experimental conditions of each experiment. Images of N2A and tsA-201 cells were obtained using a 63 x oil objective at a resolution of 1,024 × 1,024 pixels and an optical section of 0.5 μm. After choosing a region of interest containing transfected cells, the 3 × 3 tile function of the microscope allowed imaging of a larger area selected without bias. Every cell identified as transfected was included in the measurements, to ensure lack of bias.

Images of tsA-201 and N2A cells were analyzed using imageJ (*imagej.net)*. Surface labeling in non-permeabilized cell bodies was measured using the freehand line tool (5 pixels) to measure the mean intensity of cell-surface staining. Intracellular staining was measured using the freehand selection tool, excluding the nucleus and the plasma membrane. The value of the mean pixel intensity in different channels was measured separately and background was subtracted by measuring the intensity of an imaged area without transfected cells. All data were then normalized to the appropriate positive control for each experiment before combining experiments.

Hippocampal neurons were imaged using a 20 x objective with a 5 μm optical section, to capture neurites (Figures 5C and 5E). Neurons expressing mCherry were selected. Acquisition settings, chosen to ensure that images were not saturated, were kept constant for each experiment. The fluorescence intensity along up to 5 neuronal projections / cell was assessed in ImageJ as follows: two concentric circles (100 and 150 μm diameter) were centered on the soma, and the freehand line tool (3 pixels) was used to trace the neuronal processes between the circles, using the mCherry image as the template. The mean gray intensity of all the pixels within the line was measured in both channels corresponding to the fluorescence of HA immunostaining and mCherry. The background fluorescence was taken in an area with no transfected cells and subtracted from the mean intensity. Mean intensity for all neurites in each neuron was calculated, and data were then averaged and presented per neuron. For high definition images of hippocampal neurons (Figure 6), fields of view were acquired using a 63 x objective (NA 1.4) with a 1 μm optical section at a resolution of 1,424 × 1,424 pixels (95 nm per pixel). For quantification in the soma of neurons, surface labeling (HA staining) was measured using the freehand line tool in imageJ (10 pixels) to measure the mean intensity. Intracellular staining (GFP fluorescence) was measured using the freehand selection tool, excluding the nucleus. For quantification in the presynaptic terminals, 2 μm diameter circular region of interests were placed on all varicosities positive for VAMP-mCherry and the mean gray intensity for GFP signal and HA staining was measured.

#### Electrophysiology

Calcium channel currents in transfected tsA-201 cells were investigated by whole cell patch-clamp recording, essentially as described previously (Berrow et al., 1997). The patch pipette solution contained in mM: Cs-aspartate, 140; EGTA, 5; MgCl_2_, 2; CaCl_2_, 0.1; K_2_ATP, 2; HEPES, 20; pH 7.2, 310 mOsm with sucrose. To reduce outward currents, we used an N-methyl-D-glucamine (NMDG)-based internal solution containing in mM: NMDG, 140; EGTA, 5; MgCl_2_, 2; CaCl_2_, 0.1; K_2_ATP, 2; HEPES, 20; pH 7.2, 310 mOsm. The external solution for recording Ba^2+^ currents contained in mM: tetraethylammonium (TEA) Br, 160; KCl, 3; NaHCO_3_, 1.0; MgCl_2_, 1.0; HEPES, 10; glucose, 4; BaCl_2_, 1,or 2 as indicated, pH 7.4, 320 mosM with sucrose. 1 mM extracellular Ba^2+^ was the charge carrier. When used, 1 μM ω-CTX was applied by local perfusion. An Axopatch 1D or Axon 200B amplifier was used, and whole cell voltage-clamp recordings were sampled at 10 kHz frequency, filtered at 2 kHz and digitized at 1 kHz. 70%–80% series resistance compensation was applied and all recorded currents were leak subtracted using P/8 protocol. Membrane potential was held at – 80 mV, unless stated. Analysis was performed using Pclamp 9 (Molecular Devices) and Origin 7 (Microcal Origin, Northampton, MA). Current-voltage (*I-V*) relationships were fit by a modified Boltzmann equation as follows: *I = G*_*max*_*^∗^(V-V*_*rev*_*)/(1+exp(-(V-V*_*50, act*_*)/k)),* where *I* is the current density (in pA/pF), *G*_max_ is the maximum conductance (in nS/pF), *V*_rev_ is the apparent reversal potential, *V*_50, act_ is the midpoint voltage for current activation, and *k* is the slope factor. The amplitude of tail currents was measured from the current present upon repolarization to −50 mV from a 20 ms test pulse between −50mV and +70 mV from −100mV holding potential. The activation curve obtained from tail currents was fitted with Boltzmann function.

### Quantification and Statistical Analysis

Data were analyzed with GraphPad Prism 7 (GraphPad software, San Diego, CA) or Origin-Pro 2015 (OriginLab Corporation, Northampton, MA, USA). All data are shown as mean ± SEM; “n” refers to number of cells, unless indicated otherwise, and is given in the figure legends, together with details of statistical tests used. Experiments where representative data are shown were repeated at least 3 times, unless otherwise stated. Graphpad Prism 7 was used for statistical analysis. Statistical significance between two groups was assessed by Student’s t test, as stated. One-way ANOVA and the stated post hoc analysis was used for comparison of means between three or more groups.

### Data and Code Availability

This study did not generate/analyze datasets or code.

## Supplemental Information

Document S1. Figures S1–S6 and Tables S1 and S2

Document S2. Article plus Supplemental Information
