# Supplementary material for: Disruption of the Key Ca2+ Binding Site in the Selectivity Filter of Neuronal Voltage-Gated Calcium Channels Inhibits Channel Trafficking
Source: Cell Rep. 2019 Oct 1;29(1):22–33.e5. doi: 10.1016/j.celrep.2019.08.079 (PMC6899504; doi:10.1016/j.celrep.2019.08.079)
Supplement: Document S1. Figures S1–S6 and Tables S1 and S2 [file mmc1.pdf]

Cell Reports, Volume *29*

Supplemental Information

Disruption of the Key Ca^{2+} Binding Site in the Selectivity Filter of Neuronal Voltage-Gated Calcium Channels Inhibits Channel Trafficking

James O. Meyer, Shehrazade Dahimene, Karen M. Page, Laurent Ferron, Ivan Kadurin, Joseph I.J. Ellaway, Pengxiang Zhao, Tarun Patel, Simon W. Rothwell, Peipeng Lin, Wendy S. Pratt, and Annette C. Dolphin

Supplemental Information for Meyer et al

Supplemental Methods

Supplemental Table S1 (relates to all Figure parts in column 1: Fig. 1, 2, 7, S6): Fit parameters for IV curves

Data		$V_{50, act}$ (mV)	G_{max} (nS.pF^{-1})	V_{rev} **(apparent)** (mV)
Fig. 1C	WT $Ca_V2.2$-HA	-5.69 ± 1.12	3.87 ± 0.78	47.61 ± 1.43
Fig. 1F	WT $Ca_V2.2$-HA (tail currents)	+2.95 ± 0.52	N/A	N/A
Fig. 2B	WT $Ca_V2.2$-HA	-16.20 ± 0.78	1.44 ± 0.14	37.71 ± 0.58
	$Ca_V2.2$-HA E_IA	-12.24 ± 1.36	0.73 ± 0.12	21.60 ± 1.98
	$Ca_V2.2$-HA $E_{IV}A$	-11.97 ± 1.06	0.57 ± 0.15	33.65 ± 1.10
Fig. 2E	WT $Ca_V2.2$-HA	-7.19±0.27	1.59±0.04	48.23 ± 0.61
Fig. 7B	WT $Ca_V2.1$-HA	-5.06 ± 1.06	1.23 ± 0.21	48.07 ± 1.96
Fig. S6B	WT $Ca_V2.1$	-5.71 ± 0.96	2.74 ± 0.42	45.44 ± 1.05
	WT $Ca_V2.1$-HA 37ºC	-0.72 ± 0.93	0.46 ± 0.08	49.56 ± 1.13
	WT $Ca_V2.1$-HA 30ºC	-2.49 ± 1.13	1.72 ± 0.42	47.55 ± 0.72

All data are mean ± SEM

Supplemental Table S2 (relates to Experimental Methods): Oligonucleotides used for making constructs.

Ca$_V$2.1-HA	Forward 5’ GCT ACC CGT ACG ACG TCC CGG ACT ACG CCG GCG TCG ACA TCA AAC CGG GTA CAT CCT TTG GAA TCA GCG TG 3’<br>Reverse 5’CGT AGT CCG GGA CGT CGT ACG GGT AGC CGG CGT AGT CGG GGA CGT CGT AGG GGT AGA CGG CCC AGA TGA CTT C 3’
Ca$_V$2.1-HA E$_I$A	Forward 5’ GTG CAT CAC CAT GGC AGG CTG 3’<br>Reverse 5’ CAG CCT GCC ATG GTG ATG CAC 3’
Ca$_V$2.1-HA E$_{IV}$A	Forward 3’ ATA ACT TCC GGA CCT TCT TCC AAG CTC TCA TGC TTC TCT TTC GGA GCG CCA CAG GGG CAG CGT GGC AC 3’
Ca$_V$2.1-HA E$_{I,II,III,IV}$A	Forward 5’ GAC TGG CGC GGA TTG GAA TGA GGT C 3’<br>Reverse 5’ CAA TCC GCG CCA GTC AGG ATC 3’<br>Forward 5’ GTG TCC ACG GGA GCG GGC TG 3’<br>Reverse 5’ CAG CCC GCT CCC GTG GAC AC 3’
Ca$_V$2.2-HA E$_I$A	Forward 5’ CAT CAC CAT GGC GGG CTG GAC TGA C 3’<br>Reverse 5’ CCA GCC CGC CAT GGT GAT GC 3’
Ca$_V$2.2-HA E$_{IV}$A	Forward 5’ CAC GGG GGC GGC CTG GCA CGA G 3’<br>Reverse 5’ CTC GTG CCA GGC CGC CCC CGT G 3’
Ca$_V$2.2-HA E$_{I,II,III,IV}$A	Reverse 5’ CAT GAC CGC ATT CCA GTC CGC TCC GGT CAG GAT CTG 3’<br>Forward 5’ CAC AGG GGC AGG GTG GCC CAT GGT G 3’<br>Reverse 5’ GCC ACC CTG CCC CTG TGG AC 3’
Ca$_V$2.2-HA E$_{IV}$K	Forward 5’ CAC GGG GAA GGC CTG GCA CGA G 3’<br>Reverse 5’ CTC GTG CCA GGC CTT CCC CGT G 3’

SUPPLEMENTAL FIGURES

Figure S1 (relates to Figure 1): I_{Ba} for selected $Ca_V2.2$ pore-mutant channels in tsA-201 cells

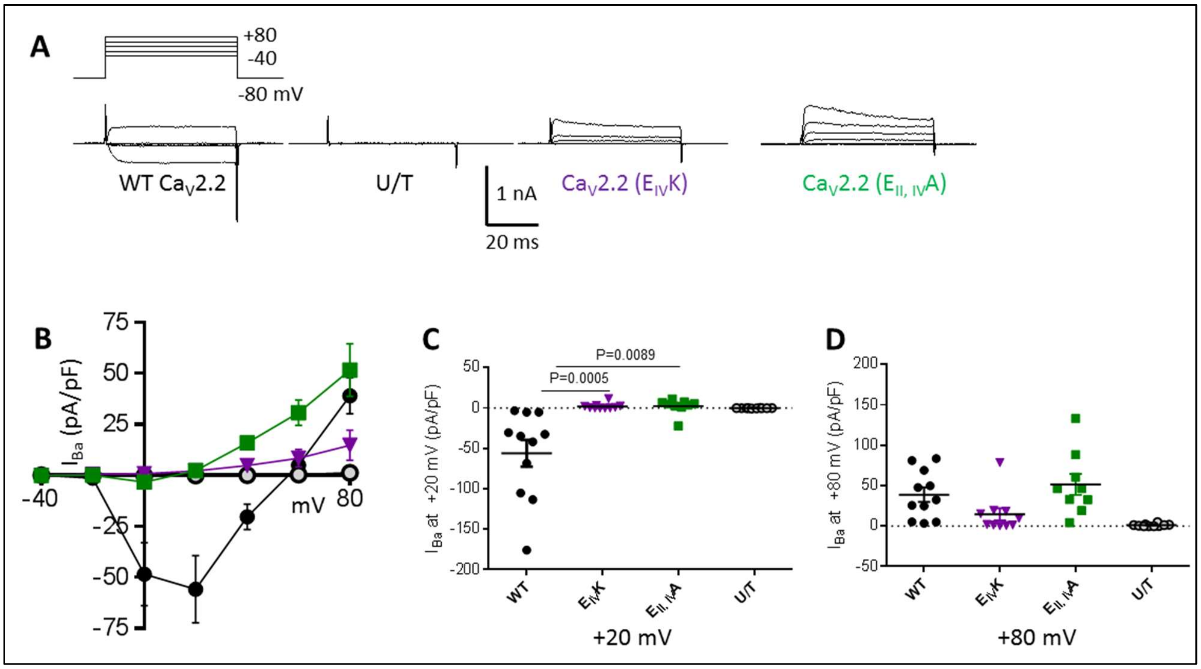

(A) Example families of $Ca_V2.2$ currents for WT $Ca_V2.2$-HA, untransfected (U/T) tsA-201 cells, $Ca_V2.2$-HA $E_{IV}K$ and $Ca_V2.2$-HA $E_{II,IV}A$, co-expressed (except U/T) with β1b-GFP and $\alpha_2\delta$-1. Holding potential -80 mV, steps between -50 and +80 mV for 50 ms in 10 mV steps (top panel, applies to all traces). Experiments used standard Cs Aspartate patch pipette solution.

(B) Mean (± SEM) *I-V* relationships for the conditions shown in (A). WT $Ca_V2.2$-HA (solid black circles, n = 11), U/T tsA-201 cells (open black circles, n = 10), $Ca_V2.2$-HA $E_{IV}K$ (solid purple triangles, n = 10) and $Ca_V2.2$-HA $E_{II,IV}A$ (solid green squares, n = 9), co-expressed with β1b and $\alpha_2\delta$-1.

(C, D) I_{Ba} at +20 mV (C) and +80 mV (D) from the *I-V* relationships shown in (B). Individual data (same symbols as B) and mean ± SEM are plotted. Statistics are obtained by 1-way ANOVA with Bonferroni's multiple comparison test, compared to WT).

Figure S2 (relates to Figures 1 and 2): I_{Ba} at +60 mV for selected $Ca_V2.2$ pore-mutant channels in tsA-201 cells

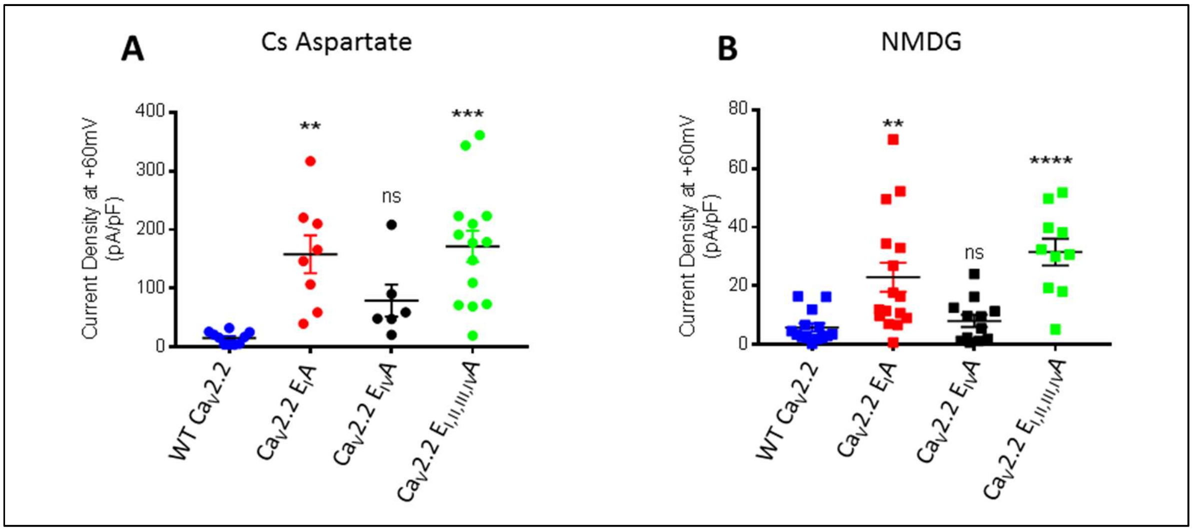

(A) I_{Ba} at +60 mV from the *I-V* relationships, using Cs Aspartate internal solution, shown in Figure 1C. Individual data (same colours as Figure 1C) and mean ± SEM are plotted. Statistics are obtained by 1-way ANOVA with Dunnett's multiple comparison correction compared to WT. ** $P = 0.0016$; *** $P = 0.0001$; ns, $P = 0.2913$.

(B) I_{Ba} at +60 mV from the *I-V* relationships, using NMDG internal solution, shown in Figure 2B. Individual data (same colours as Figure 2B) and mean ± SEM are plotted. Statistics are obtained by 1-way ANOVA with Dunnett's multiple comparison correction compared to WT. ** $P = 0.0030$; **** $P < 0.0001$; ns, $P = 0.951$.

Figure S3 (relates to Figure 3): Effect of mutations in the $Ca_V2.2$ selectivity filter on its cell surface and intracellular expression in cell lines

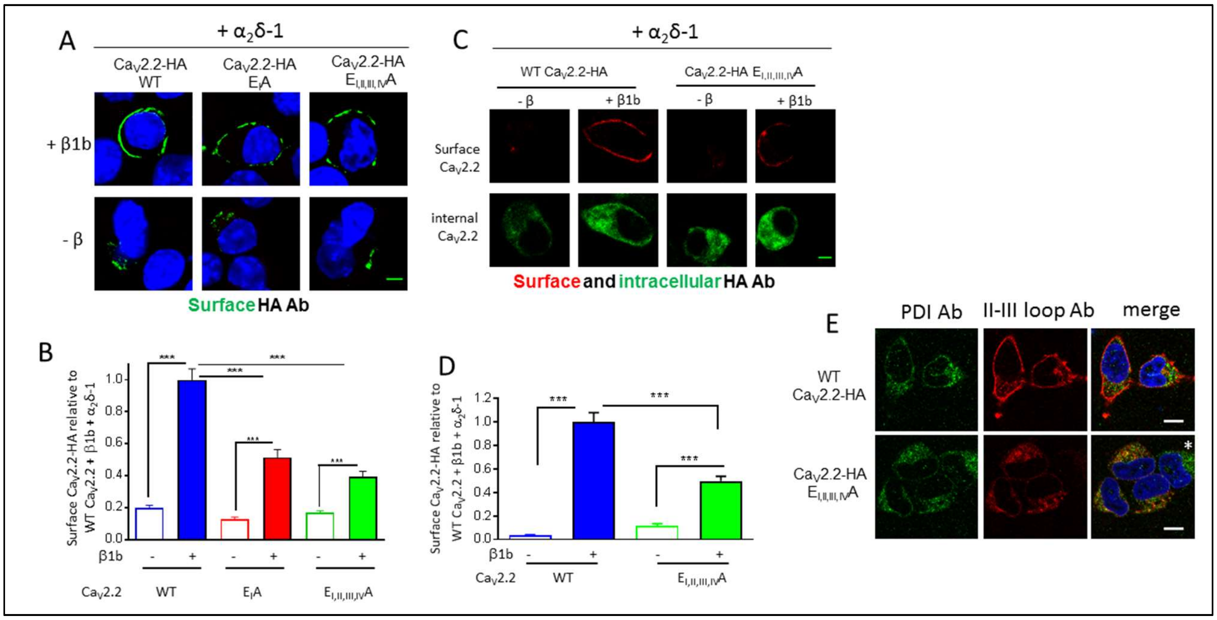

(A) Images of cell surface (HA Ab, green) WT $Ca_V2.2$-HA (left), $Ca_V2.2$-HA E_IA (middle) and $Ca_V2.2$-HA $E_{I,II,III,IV}A$ (right) in non-permeabilized N2A cells when co-expressed with $\alpha_2\delta$-1 and β1b (upper panel) or without β (lower panel). Nuclei stained with DAPI (blue). Scale bar 5 µm.

(B) Bar chart for surface $Ca_V2.2$ expression for WT $Ca_V2.2$-HA/$\alpha_2\delta$-1 (blue open; n = 116 cells) , WT $Ca_V2.2$-HA/β1b/$\alpha_2\delta$-1 (blue solid; n = 95 cells), $Ca_V2.2$-HA E_IA/$\alpha_2\delta$-1 (red open; n = 96 cells), $Ca_V2.2$-HA E_IA/β1b/$\alpha_2\delta$-1 (red solid; n = 97 cells), $Ca_V2.2$-HA $E_{I, II, III, IV}A$/$\alpha_2\delta$-1 (green open; n = 81 cells), $Ca_V2.2$-HA $E_{I, II, III, IV}A$/β1b/$\alpha_2\delta$-1 (green solid; n = 86 cells) in N2A cells. Data (mean ± SEM) were normalized to the WT $Ca_V2.2$-HA/β1b/$\alpha_2\delta$-1 condition. Statistical significance was determined using one-way ANOVA and Bonferroni post hoc tests (*** $P<0.001$). Cells were selected by increasing the gain on the images, in order to identify transfected cells. This manipulation was only used for cell selection and did not affect the data analysis. The absence of β1b produced an 80.0 % reduction for WT $Ca_V2.2$ and a 57.1 % reduction for $Ca_V2.2$-HA $E_{I, II, III, IV}A$.

(C) Images of cell surface $Ca_V2.2$-HA (red, top panel) and intracellular $Ca_V2.2$-HA (green, bottom panel) in N2A cells expressing: WT $Ca_V2.2$-HA/$\alpha_2\delta$-1 or $Ca_V2.2$-HA $E_{I, II, III, IV}A$/$\alpha_2\delta$-1 in the presence or absence of β1b. Scale bar 5 µm.

(D) Bar chart for surface $Ca_V2.2$ expression for WT $Ca_V2.2$-HA/$\alpha_2\delta$-1 (blue open; n = 106 cells), WT $Ca_V2.2$-HA/β1b/$\alpha_2\delta$-1 (blue solid; n = 115 cells), $Ca_V2.2$-HA $E_{I, II, III, IV}A$/$\alpha_2\delta$-1 (green open; n = 104 cells), $Ca_V2.2$-HA $E_{I, II, III, IV}A$/β1b/$\alpha_2\delta$-1 (green solid; n =115 cells) in N2A cells. Data (mean ± SEM) were normalized to the WT $Ca_V2.2$-HA/β1b/$\alpha_2\delta$-1 condition. Statistical significance was determined using one-way ANOVA and Bonferroni post hoc tests (***$P<0.001$). Cells were selected based on intracellular expression of HA Ab, performed following cell permeabilization (see Methods). The absence of β1b produced a 96.6 % reduction for WT $Ca_V2.2$ and a 75.9 % reduction for $Ca_V2.2$-HA $E_{I, II, III, IV}A$.

(E) Images showing protein disulfide isomerase (PDI, green, left), $Ca_V2.2$ II-III loop Ab staining (red, middle) and merged images (right, showing co-localization in yellow) for WT $Ca_V2.2$-HA (upper panel) and $Ca_V2.2$-HA $E_{I,II,III,IV}A$ (lower panel) co-expressed with $\alpha_2\delta$-1 and β1b in tsA-201 cells. Nuclei are stained with DAPI in the merged image (blue). The asterisk (*) indicates a cell labelled with PDI but untransfected, showing the specificity of the II-III loop Ab. Scale bars 10 µm.

Figure S4 (relates to Figure 6): Properties of Ca$_V$2.2 pore mutant channels with an N-terminal GFP tag

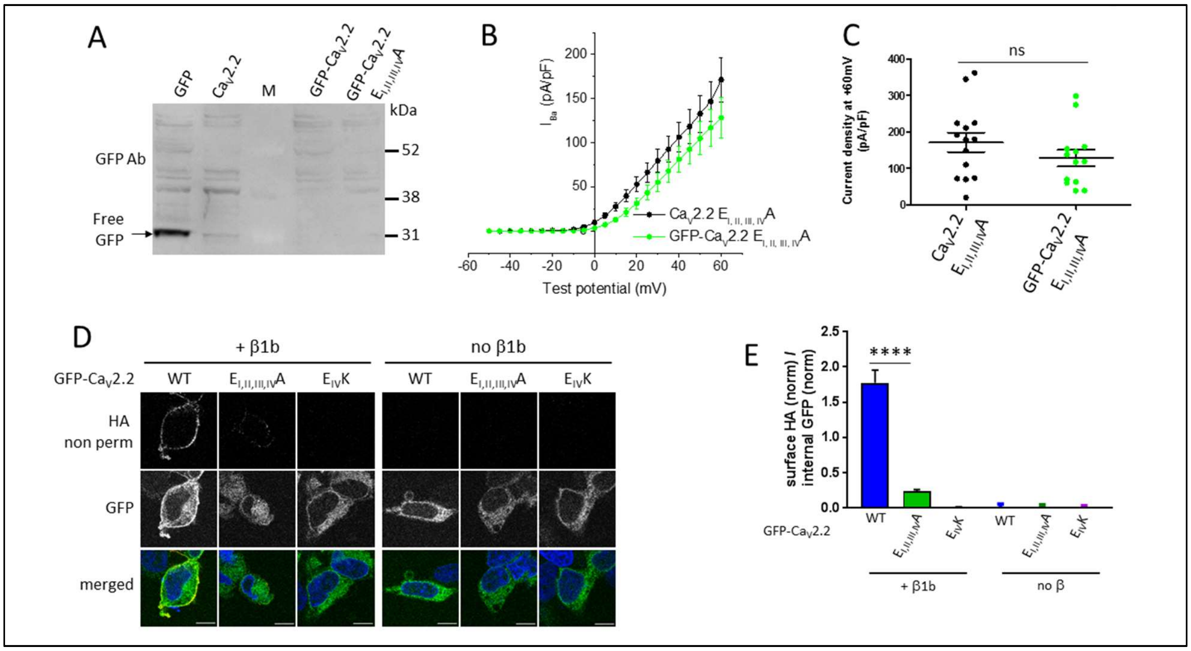

(A) Western blots using polyclonal GFP Ab for (left to right) free GFP, WT Ca$_V$2.2, GFP-Ca$_V$2.2 and GFP-Ca$_V$2.2 $E_{I,\ II,\ III,\ IV}$A, expressed in N2A cells. M = MW marker lane.

(B) Mean (± SEM) I-V relationships for Ca$_V$2.2-HA $E_{I,\ II,\ III,\ IV}$A (solid black circles, n=16) and GFP-Ca$_V$2.2-HA $E_{I,\ II,\ III,\ IV}$A (solid green circles, n=13), co-expressed with $\alpha_2\delta$-1 and β1b.

(C) I_{Ba} at +60 mV from the *I-V* relationships in (B), Individual data (same colours as in B) and mean ± SEM are plotted. (ns = non-significant, Student's t test).

(D) Representative images of cell surface expression of GFP-Ca$_V$2.2-HA (HA Ab, top row) in non-permeabilized tsA-201 cells of (left to right) WT GFP-Ca$_V$2.2-HA, GFP-Ca$_V$2.2-HA $E_{I,\ II,\ III,\ IV}$A and GFP-Ca$_V$2.2-HA E_{IV}K in the presence of β1b (first three columns) or the absence of β (right three columns). All conditions contained $\alpha_2\delta$-1. Second row shows GFP fluorescence of the GFP tag. Third row shows merged images with co-localization of HA and GFP in yellow, GFP in green and nuclei stained with DAPI (blue). Scale bar 10 µm.

(E) Ratio of tsA-201 cell surface expression (measured by HA staining in non-permeabilized cells) / intracellular expression (measured by intracellular GFP), for WT GFP-Ca$_V$2.2-HA (blue bar, n = 364 cells), GFP-Ca$_V$2.2-HA $E_{I,\ II,\ III,\ IV}$A (green bar, n = 391 cells) and GFP-Ca$_V$2.2-HA E_{IV}K (violet bar, n = 368 cells), co-expressed with $\alpha_2\delta$-1 and β1b. The effect of lack of β-subunit is also shown, for WT GFP-Ca$_V$2.2-HA (n = 231 cells), GFP-Ca$_V$2.2-HA $E_{I,\ II,\ III,\ IV}$A (n = 249 cells) and GFP-Ca$_V$2.2-HA E_{IV}K (n = 166 cells), all co-expressed with $\alpha_2\delta$-1 in the absence of β1b. Data (mean ± SEM) from 3 separate experiments. Statistical significance was determined using one-way ANOVA and Bonferroni post hoc test (**** P<0.0001).

Figure S5 (relates to Figure 6): Cell surface expression of WT Ca$_V$2.2 and selectivity filter mutant at the plasma membrane in hippocampal neuronal cell bodies

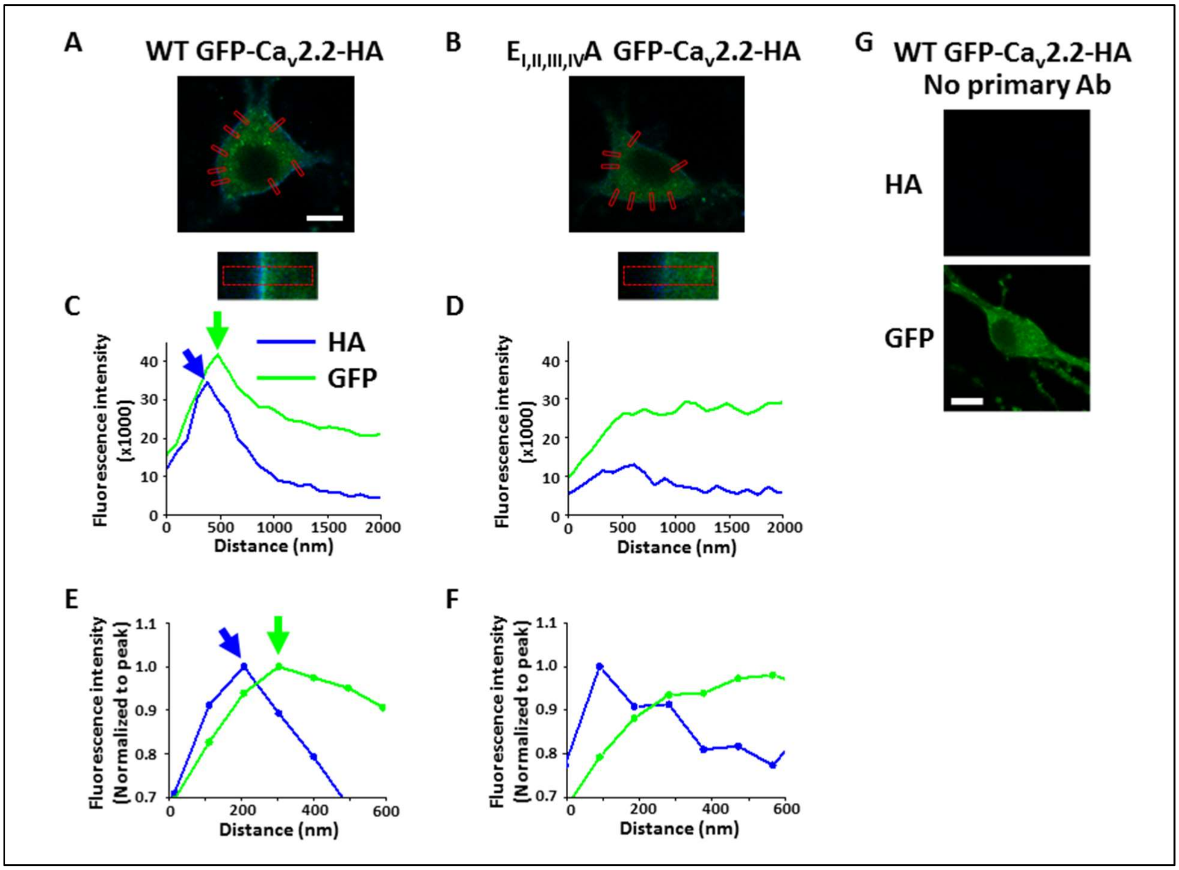

(A-B) Confocal images of soma of hippocampal neuron expressing either WT (A) or $E_{I,II,III,IV}A$ mutant (B) GFP-Ca$_v$2.2-HA channels, together with β1b and $\alpha_2\delta$-1. GFP-Ca$_v$2.2-HA was immunostained using a rat anti-HA Ab in non-permeabilizing conditions and is shown in blue. GFP fluorescence from GFP-Ca$_V$2.2-HA is shown in green. Red boxes correspond to 5 µm x 1 µm regions of interest used for line scanning across the plasma membrane. Scale bar, 10 µm. Bottom panels represent zoomed area of the plasma membrane: red dashed boxes used for line scans are 5 µm x 1 µm in size. The left part of the box corresponds to the extracellular space and the right part to the intracellular compartment.

(C-D) Fluorescence profile across the plasma membrane for HA staining and GFP for WT (C) and $E_{I,II,III,IV}A$ mutant (D) Ca$_V$2.2. Average of 8 line scans from image shown in A and B. HA staining is shown in blue and GFP fluorescence from GFP-Ca$_v$2.2-HA is shown in green. The arrows point to the peaks of fluorescence intensity (blue, HA staining; green, GFP fluorescence).

(E-F) Normalized fluorescent profile across the plasma membrane for HA staining (blue) and GFP (green) for WT (E) and $E_{I,II,III,IV}A$ mutant (F) Ca$_V$2.2. Average of 5 to 6 cells. Data for each fluorescent profile were normalized to the peak of fluorescent intensity. The arrows point to the peaks of fluorescence intensity (blue, HA staining; green, GFP fluorescence). Peaks for HA staining and GFP fluorescence are 100 nm apart.

(G) Confocal images of soma of a hippocampal neuron expressing WT GFP-Ca$_v$2.2-HA channels. GFP-Ca$_v$2.2-HA was immunostained omitting the primary Ab, in non-permeabilizing conditions (an anti-rat AF-647 Ab was used as a secondary Ab, upper image). GFP fluorescence from GFP-Ca$_v$2.2-HA is shown in green (lower image). Scale bar, 10 µm.

Figure S6 (relates to Figure 7): Expression of Ca$_V$2.1-HA in tsA-201 cells

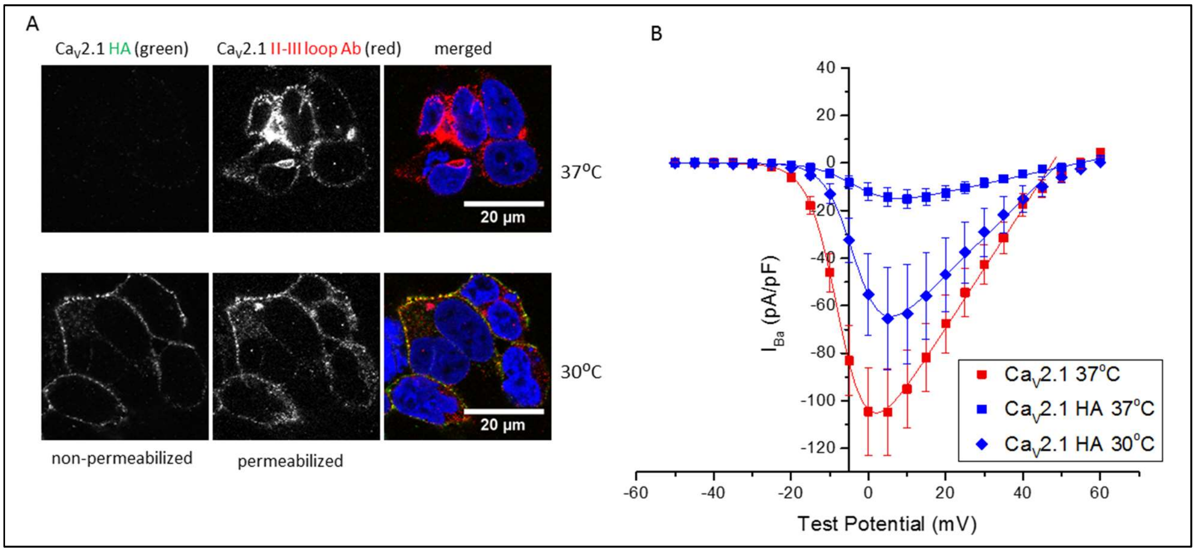

(A) Ca$_V$2.1-HA, β1b and $\alpha_2\delta$-1 were expressed in tsA-201 cells, which were either maintained at 37 °C following transfection (upper row), or with a period at 30 °C (lower row), as described in Methods. Left panel: cell surface HA signal in non-permeabilized cells; middle panel: Ca$_V$2.1 II-III loop signal following permeabilization; right panel: merged images, surface HA in green, total II-III loop staining in red, co-localization in yellow. The nuclei are stained with DAPI (blue). Scale bar: 20 µm.

(B) Mean (± SEM) *I-V* relationships for WT Ca$_V$2.1 (cells maintained at 37 °C; solid red squares, n = 22), Ca$_V$2.1-HA (cells maintained at 37 °C; solid blue squares, n = 16) and Ca$_V$2.1-HA (cells maintained at 30 °C; solid blue diamonds, n = 8). The mean data in (B) were fit with a modified Boltzmann relationship (solid line). Fit data in Supplementary Table 1.
